# Effects of Production Region, Production Systems and Grape Type/Variety on Nutritional Quality Parameters of Table Grapes; Results from a UK Retail Survey

**DOI:** 10.3390/foods9121874

**Published:** 2020-12-16

**Authors:** Gultakin Hasanaliyeva, Eleni Chatzidimitrou, Juan Wang, Marcin Baranski, Nikolaos Volakakis, Chris Seal, Eduardo A. S. Rosa, Per Ole Iversen, Vanessa Vigar, Bronwyn Barkla, Carlo Leifert, Leonidas Rempelos

**Affiliations:** 1 Department of Sustainable Crop and Food Protection, Faculty of Agriculture, Food and Environmental Sciences, Universita Catollica del Sacro Cuore, I-29122 Piacenza, Italy; gultekin.hasanaliyeva@gmail.com; 2 Nafferton Ecological Farming Group, School of Agriculture, Food and Rural Development, Newcastle University, Newcastle upon Tyne NE1 7RU, UK; eleni.e.chatzidimitriou@gmail.com (E.C.); j.wang28@outlook.com (J.W.); leonidas.rempelos@newcastle.ac.uk (L.R.); 3 French Agency for Food, Environmental and Occupational Health and Safety (ANSES), Regulated Products Assessment Department, Residues and Food Safety Unit, 14 rue Pierre et Marie Curie, 94701 Maisons-Alfort, France; 4 Human Nutrition Research Centre, Human Nutrition Research Centre, Institute of Cellular Medicine, Newcastle University, Newcastle upon Tyne NE2 4HH, UK; chris.seal@newcastle.ac.uk; 5 School of Agriculture and Biology, Shanghai Jiao Tong University, Shanghai 200240, China; 6 Department of Functional and Organic Food, Institute of Human Nutrition Sciences, Warsaw University of Life Sciences, Nowoursynowska 159c, 02-776 Warsaw, Poland; m.baranski@nencki.edu.pl; 7 Geokomi plc, Agriculture Consultancy, P. O. Box 21, Sivas-Faistos, GR 70200 Crete, Greece; nvolakakis@gmail.com; 8 Centre for the Research and Technology of Agro-Environment and Biological Sciences, Universidade de Trás-os-Montes e Alto Douro (UTAD), 5001-801 Vila Real, Portugal; erosa@utad.pt; 9 Department of Nutrition, Institute of Basic Medical Sciences, University of Oslo, 0372 Oslo, Norway; p.o.iversen@medisin.uio.no; 10 Department of Haematology, Oslo University Hospital, 0372 Oslo, Norway; 11 Centre for Organics Research, Southern Cross University, Military Road, Lismore, NSW 2480, Australia; Vanessa.Vigar@scu.edu.au; 12 Southern Cross Plant Science, Southern Cross University, Military Road, Lismore NSW 2480, Australia; bronwyn.barkla@scu.edu.au

**Keywords:** table grapes, phenolics, anthocyanins, antioxidant activity, TEAC, DPPH, organic, conventional

## Abstract

Grapes contain high concentrations of secondary metabolites and antioxidants that have been linked to a reduction of several chronic diseases. Here, we report results of a UK retail survey, which investigated the effect of the production region (Mediterranean vs. South Africa), grape type (white vs. red vs. black) and variety, and production system (organic vs. conventional) on antioxidant activity and concentrations of phenolic compounds in table grapes. Black grapes had ~180% total antioxidant activity (TAA), ~60% higher total phenolic content (TPC) and ~40 times higher anthocyanin concentrations (TAC) than white grapes, while red grapes had intermediate levels of TAA, TPC and TAC. The effects of season and production system and differences between varieties of the same grape type were substantially smaller. Grapes imported from Mediterranean countries in summer had a 14% higher TPC and ~20% higher TAA than grapes imported from South Africa in winter, and organic grapes had a 16% higher TPC and 22% higher TAA, but ~30% lower TAC than conventional grapes. Significant differences in TPC, TAA and/or TAC between organic and conventional grapes could only be detected for specific grape types, varieties and/or sampling years.

## 1. Introduction

Grapes are one of the most popular fruit species globally and are used as table grapes and for processing into grape juice, wine and a range of other alcoholic drinks (e.g., brandy, raki, grappa). FAO [1] statistics suggest that around 76,000 square kilometers of agricultural land is used for grape production and approximately 21% of global grape production is for table grapes. Table grapes consumed in Europe are mainly produced in (a) Mediterranean countries (summer grape season) and (b) countries in the Southern Hemisphere (mainly South Africa and to a lesser extent Australia, Argentinia, Brazil, Chile and New Zealand) [2].

Grapes have high concentrations of plant metabolites with antioxidant activity including polyphenols, such as flavonoids, tannins, resveratrol and anthocyanins [3,4,5]. There is mounting scientific evidence for positive links between consumption of antioxidant/polyphenol-rich foods/drinks and lower risk of cardiovascular and other chronic diseases [6,7]. For example, a large epidemiological study that monitored diet and health of human participants over 20 years has described the consumption of grapes and grape products (in particular red wine) as a possible explanation for the “French paradox”, the observation that French consumers, despite consuming high fat/animal fat diets, have a very low rate of coronary heart disease [8,9]. However, this and previous studies showed that total antioxidant activity and total concentrations and profiles of individual phenolics, in particular anthocyanins, differ substantially between different grape types (white, red and purple/black), with levels often shown to be lowest in white and highest in black grapes [10]. It is therefore likely that the type of grape consumed may also affect the relative impact of grape consumption on health.

The demand for organic table grapes has rapidly increased mainly due to consumer perceptions that organic foods have higher sensory and nutritional quality [11,12,13]. A recent systematic review and meta-analysis reported that organic crops on average have higher concentrations of phytochemical/antioxidants and higher antioxidant activity [14]. Long-term field experiments on arable crops linked the higher phenolic concentrations and antioxidant activity in organic compared to conventional wheat to a lower and/or more balanced supply of nitrogen when organic instead of mineral N-fertilizers are used [15]. However, there is still considerable controversy on whether and to what extent organic table grapes have higher phenolic, anthocyanin and/or antioxidant levels, because several previous comparative studies, reported contrasting results on composition differences between organic and conventional table grapes or grapes of specific varieties [12,16,17,18,19,20,21,22]. For example, a study by Tassoni et al. [22,23] reported significantly higher total antioxidant (TAA_TEAC_) levels in (a) organic white grapes of the variety Albana, (b) organic red grapes of the variety Lambrusco, but (c) conventional red grapes of the variety Sangiovese, while production system had no significant effect on TAA_TEAC_ in white grapes of the variety Pignoletto.

Apart from variety choice and environmental conditions (e.g., temperature, solar radiation, soil type), a range of specific agronomic factors (including fertilization, irrigation, use of plant growth regulators and bud load levels, cluster thinning and fruit yield) factors are known to affect total phenolic, anthocyanin and/or antioxidant activity levels in gapes, and may therefore explain the variability of results of these comparative (organic vs. conventional) studies.

The contrasting effects of grape exposure to high temperatures (>35 °C) and solar irradiation were studied in a groundbreaking study by Tarara et al. [24]. Total anthocyanin content in grape skins decreased when berries were exposed to temperatures above 35 °C, while berries exposed to sun had more than twice the amount of flavonol-glycosides than berries from shaded clusters [24].

The type of fertilizer, input levels, and the ratio (e.g., N:K) of mineral fertilizers applied to grapes are shown to affect phenolic and/or antioxidant levels in grapes [25,26,27]. Previous studies showed that the ratio of N:K applied with fertilizer is an important driver for grape polyphenol and anthocyanin levels. For example, a study by Delgado et al. [25] reported that increasing mineral N-applications in combination with no or medium-level K fertilizer reduce polyphenol and anthocyanin concentrations in Tempranillo grapes, while grapes fertilized with high levels of both mineral N and K fertilizer had the highest polyphenol concentrations. Fruit with an N:K ratio of between 3.6 and 4.3 were shown to have the highest polyphenol levels [25]. Similar trends were found in a long-term study in France which compared different input levels of cattle manure, with high levels of manure inputs resulting in lower anthocyanin and tannin content in grapes, but interestingly higher anthocyanin levels in wine made from them [26]. A recent study from Iran reported lower fruit yields, but higher antioxidant activity and phenolic levels when white seedless grapes were fertilized with organic than mineral fertilizers [27].

Studies by Peterlunger et al. [28] with Merlot grapes in Italy and Kennedy et al. [29] with Savignon grapes in the US both reported that, compared to an optimum irrigation control, minimum/deficit irrigation treatment resulted in higher anthocyanin concentrations.

A study in Spain with Tempranillo and Grenache grapes reported that mechanical thinning led to a higher Brix and increased concentrations of berry phenolics, but not anthocyanins and the study also found strong treatment x year interactions for berry phenolics [30].

The use of gibberellic acid (GA_3_) to increase fruit yield was reported to decrease total phenolic, flavonoid and antioxidant activity in ‘Muscat’ grape berry skin and flesh, although levels in leaf, stem and tendril tissue increased [31]. Iincreasing high yield levels by contrasting levels of winter pruning or cluster thinning were also reported to result in lower total phenolic, anthocyanin and/or antioxidant activity in table grapes, and wine [30,32,33]. Garrido et al. [33] also reported that the increase in phenylpropanoids and flavonoids concentrations effect resulting from cluster thinning/lower yields was greater in non-irrigated than irrigated grapes [11].

The main objective of the retail survey reported here was to quantify and compare the concentrations of phenolics, anthocyanins and antioxidant activity in organic and conventional white, red and/or black grapes available to consumers in UK supermarkets. Another objective was to identify composition differences between grapes from the two different regions that supply the UK and European market by comparing grapes in the two periods when both organic and conventional grapes are available in UK supermarkets, which are between (a) July and October (when grapes imported from Mediterranean countries) and (b) January and April (when grapes are imported from South Africa and other countries in the Southern hemisphere).

Preliminary supermarket surveys had shown that the varieties available as organic and conventional tables grapes differed substantially in both the winter and summer periods and variety-matched samples of organic and conventional grapes are rarely available. It was therefore not possible to quantify to what extent the contrasting variety of profiles of organic and conventional grapes contributed to/confounded the composition differences detected between organic and conventional grapes. However, to identify the potential impact of variety choice we carried out supplementary statistical analyses using data obtained for varieties available both as organic and conventional grapes only.

It should also be pointed out that, although retail surveys can provide an accurate estimate of composition differences between (a) organic and conventional and (b) white, red and black grapes experienced by consumers, they do not allow these differences to be linked to contrasting (a) pedoclimatic conditions in grape-producing regions, (b) agronomic practices (e.g., variety choice, and fertilization, pruning, crop protection regimes) and (c) supply chain-related parameters (e.g., maturity stage at harvest, postharvest treatments, time spend in transport). This is primarily because the information on pedoclimatic, agronomic and supply chain-related parameters cannot be obtained from the supermarkets where grapes were collected.

## 2. Materials and Methods

### 2.1. Retail Survey Strategy

The table grape retail survey was conducted within two seasons (winter and summer) and during two years (2015 and 2016). During each season grapes were collected at regular weekly intervals in stores of three different UK supermarkets that sold both organic and conventional table grapes within the Newcastle upon Tyne (NE) UK postcode area. Table grapes collected during the winter season were all imported from South Africa while those collected during the summer season were imported from Egypt, Morocco, Greece, Italy and Spain. Depending on the season and sampling date, different color (white, red and black) grape varieties were available in the shops. The varieties available as organic and conventional table grapes also differed on most sampling dates. White and red color grape varieties were available from both organic and conventional production in both seasons (winter and summer), whereas black colored grape varieties were only available during the summer season.

In each of the three supermarkets, all organic table grape varieties available on sampling days were purchased, together with either (a) conventional table grapes of the same variety (when available) with the same sell-by date (+/− 1 day), or (b) conventional table grapes of a different variety (if the same variety was not available), but the same color type and sell-by date (+/− 1 day).

We collected up to three paired samples (one organic and one conventional bunch/pack per supermarket) per grape type (white, red and black) on an individual sampling date (3 supermarkets × 2 production systems × 3 grape types = 18 samples). However, on many sampling dates, matching samples for some grape types were only available in one or two of the supermarkets, and black organic grapes were mainly available in the summer season when grapes were imported from the Mediterranean. A list of varieties available in the UK as both organic and conventional products in the UK during the summer and winter seasons is shown in Table 1.

### 2.2. Grape Sample Storage and Preparation

All 485 fresh grape samples (each sample being 1.5 or 2 bunch(es) of grapes in a plastic container) were transferred to Newcastle University within an hour of purchase and stored at −20 °C until sample preparation. After defrosting for 2 h at room temperature and under a dim light, about 40–50 whole grape berries (approximately 250–350 g) were randomly selected from each sample (1.5 or 2 bunch(es) of grapes/box), cut in half in order to allow removal of seeds and then crushed/homogenized (skin and pulp) for 30–120 s in a homogenizer (multipurpose food blender) to prepare grape juice. Following homogenizing, 5 aliquots of juice (for each sample) were labeled (date, management, supermarket, production country and cultivar name) and stored at −80 °C until use in different biochemical assays/analyses. Dry matter (DM) [34] and sugar content (SC) (OPTi Brix 54 Handheld Digital Refractometer) were determined as physical properties of grape fruit. SC was also included as a marker for the ripening stage at harvest [35].

### 2.3. Chemical Reagents

Folin-Ciocalteau phenol reagent, gallic acid, potassium persulfate and radical scavenging assay reagents: 6-hydroxy-2, 5, 7, 8-tetramethylchroman-2-carboxylic acid (Trolox), 2,2-diphenyl-1-picrylhydrazyl (DPPH), 2, 2′-azinobis-(3-ethylbenzothiazoline)-6-sulfonic acid (ABTS) were purchased from Sigma-Aldrich (St. Louis, MO, USA). Sodium carbonate, methanol, hydrochloric acid (12 N), sodium chloride, sodium dihydrogen phosphate, sodium hydrogen phosphate, potassium chloride, sodium acetate, formic acid, acetonitrile and methanol (for HPLC grade) were supplied by Fisher Scientific.

### 2.4. Extraction of Secondary Metabolites from Table Grapes

Secondary metabolites were extracted according to Tassoni, et al. [22]. In brief half a gram (0.5 g FW) of the homogenized grape sample was mixed with 4 mL of extraction solution (MeOH:HCl (98:2)) and left for overnight extraction under the dim light. Extracted samples then were centrifuged at 4000 rpm for 15 min at 4 °C and diluted with extraction solution.

### 2.5. Determination of Total Phenolic Content (TPC) and Total Antioxidant Activity (TAA)

Total phenolic content was determined by the Folin-Ciocalteau (FC) colorimetric assay method [36]. An extract aliquot of 20 μL was mixed with 100 μL FC solution and kept for 5 min at room temperature. After this, 300 μL of SC solution was added, covered with parafilm and left for 2 h at room temperature. The absorbance of samples was recorded at 765 nm wavelengths using a UV-VIS spectrophotometer (UV mini-1240, Shimadzu). Gallic acid (GA) solution was used as the standard for calibration curve calculations and the results were expressed as mg GA equivalent/kg of sample’s fresh weight (FW).

Total antioxidant activity was determined by the 2,2-diphenyl-1-picrylhydrazyl (DPPH) [37] and 2, 2′-azinobis-(3-ethylbenzothiazoline)-6-sulfonic acid radical (TEAC) assays [38]. Absorbance was read at 517 nm for DPPH and 734 nm for TEAC. Trolox (6-hydroxy-2, 5, 7, 8-tetramethylchroman-2-carboxylic acid) solution was used as the standard for calibration curve calculations and the results were expressed as mmol Trolox Equivalent (TE)/g of sample’s FW.

### 2.6. Extraction and Determination of Total Anthocyanin Content (TAC)

Anthocyanins were extracted from the grape sample using the methods described by Tassoni, et al. [22] and Chiou, et al. [39] with slight modifications, and the total anthocyanin content (TAC) in extracts was measured using the pH differential method [40]. In brief, half a gram (0.5 g (FW)) of the homogenized grape sample was mixed with 4 mL of 0.1% acidified methanol solution and incubated in a water bath at 65 °C for 2 h, under dim light. After incubation samples were centrifuged at 4000 rpm for 10 min, at 25 °C and diluted with pH buffers. A suitable volume of diluted grape samples was used for spectrophotometric analyses, in order to measure absorbance at 520 nm (A520) and 700 nm (A700) wavelength. Final absorbance was calculated according to the formula: A = (A520 nm − A700 nm) × pH 1.0 − (A520 nm − A700 nm) × pH 4.5. Two of the most common anthocyanin pigments (cyandin 3-glucoside and malvidin 3-glucoside) were used in the calculation as an equivalent.

### 2.7. Identification and Quantification of Individual Anthocyanins by HPLC

Individual anthocyanins in grape samples were detected and their concentrations were quantified according to Kammerer, et al. [41] with slight modifications. In brief, aliquots of 0.5 g of grape juice sample were mixed with 1.5 mL of 0.1% acidified methanol and vortexed for 2 h for complete extraction. Vortex tubes were then centrifuged at 10,600 rpm for 5 min and the supernatant was transferred into a second tube. The extraction was repeated adding 0.5 mL of 0.1% acidified methanol into the remaining residue and vortex for another 15 min. The extracts were centrifuged and the supernatants were combined and centrifuged again. After centrifugation, extracts were passed through filtered and stored at −80 °C until they were directly injected into the HPLC.

Analyses and separation of individual anthocyanin components were performed using a Phenomenex, Synergiᵀᴹ 4 μm Hydro-RP 80Å (C18 phase, 250 × 4.6 mm) column, fitted with a C18 guard column (3.2–8.0 mm internal diameters) at a temperature of 25 °C. The HPLC system (Shimadzu Corporation, Japan) was equipped with LabSolution software, a DGU-20A3R degasser, 2 LC-20AD pump, a SIL-20AC HT autosampler, a SPD- M20A diode array detector and a CTO- 20AC column oven. The detector was set to an acquisition range of 190–700 nm.

Water/formic acid/acetonitrile (A) (87:10:3) and water/formic acid/acetonitrile (B) (40:10:50) were used as a mobile phase with a flow rate of 0.8 mL/min. The gradient programme for the mobile phases (A:B) was at 0.02 min (10:90), 5 min (10:90), 15 min (25:75), 20 min (31:69), 25 min (40:60), 35 min (50:50), 45 min (100:0), 50 min (10:90) and 55 min (10:90). The injection volume was 50 μL for all samples and quantification was performed at 520 nm.

Identification of individual anthocyanins was based on peak relative retention times and elution order of chromatograms obtained by Kammerer, et al. [41]. Individual anthocyanins were quantified using a calibration curve of malvidin-3-*O*-glucoside in the range of 50 to 0.05 µg/mL. LC-MS analysis was performed separately by Newcastle University Protein and Proteome Analysis (NUPPA) laboratory team to confirm the identity of the peaks based on m/z identified by HPLC analysis.

### 2.8. Statistical Analysis

Principle component analyses (PCA) were carried out for both secondary metabolite, antioxidant activity and sugar content data obtained for individual samples using the ‘prcomp’ function in R and visualized by using the ‘autoplot’ function of the ggplot2 package in R [42]. PCA-results were then used to select appropriate Analyses of Variance (ANOVA) tests to further investigate main effects and interactions between the factors grape type (white, red, black), grape variety, management system (organic, conventional) and production region/season (South Africa, Mediterranean) included in the retail survey.

ANOVA derived from Linear mixed-effects (lme) models [43] was used to assess the effects and interactions between factors on measured parameters by using the ‘nlme’ package in R [42]. Data obtained for 410 matched samples (=organic and conventional grapes of the same grape type collected on the same date) from different sampling dates within the same year and season were used as replicates and supermarket was included as a random factor.

**Figure 1:**
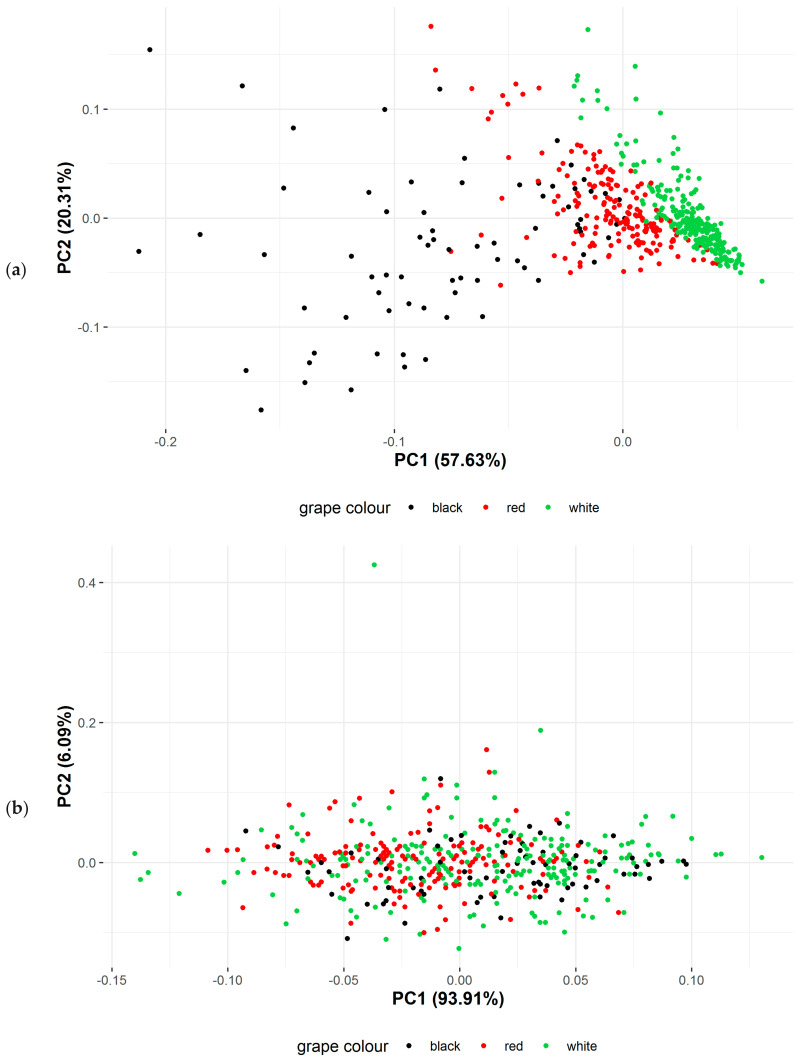
Principle component analyses of data for (**a**) secondary metabolite concentration and antioxidant activity and (**b**) sugar content (Brix) data in different grape varieties showing the level of separation/variation between grape types (white, red, black). Dots are results obtained for individual grape samples.

For secondary metabolites and antioxidant activity, PCA identified a clear separation between results obtained for different grape types (white, red, black) (Figure 1a), but not year, production region or production system (Figure S1). For sugar content no clear separation of data was detected between different years, production regions, production systems and grape types (Figure 1b and Figure S2).

The two main factorial ANOVA, therefore, focused on identifying the main effects of grape type and potential interactions between grape type, and year, production region and production system. ANOVA 1 was based on all data from matching organic and conventional white and red grape samples and included (a) year, (b) production system (organic vs. conventional), (c) season (winter vs. summer) and (d) grape type (white vs. red) as factors. ANOVA 2 was based on all data from matching organic and conventional white, red and black samples collected in the summer season.

The hierarchical nature of the experimental design was designated in the random error structures of the model as: replicate (brand)/year/production system/season (ANOVA 1); and replicate (brand)/year/production system (ANOVA 2).

Separate PCA with data obtained for white (Figure S3), red (Figure S4) and black (Figure S5) grapes were also carried out to identify separation/variation between (a) different varieties of the same grape type (b) grapes of the same variety produced by organic and conventional production systems. PCA results indicated some separation/variation between varieties for all three grape types and for some varieties also between organically and conventionally produced grapes.

However, due to the differences in the range of varieties available (1) in summer and winter and (2) from organic and conventional production systems (Table 1), variety could not be included as an additional factor in the main ANOVA described above. In order to identify/estimate potential confounding effects of variety choice we, therefore, carried out separate supplementary ANOVA for subsets of data from (1) white, (2) red and (3) black varieties that were available both (a) from organic and conventional production and/or (b) in summer and winter. Detailed results of the supplementary ANOVA are provided in the supplementary information only (Tables S1–S9), because most comparisons are based on (a) a low number of replicates and (b) data from only one year and/or production region, and therefore need to be interpreted carefully until they are confirmed in future studies.

In order to further investigate the significant (*p* < 0.05) interactions between factors, general linear hypothesis tests (Tukey contrasts) were performed using the ‘glht’ function of the ‘multcomp’ package in R [44]. The experimental design was reflected in the same random error structures used for the lme models. This method allows multiple comparisons in unbalanced models with arbitrary error distribution and hence arbitrary data distribution and variance structure. Real means and standard errors of means for the main effect and the interaction tables were generated by using the ‘tapply’ function in R.

## 3. Results

The retail survey reported here was carried out to compare the nutritional quality of different table grapes products available to consumers via supermarket supply chains. Samples of organic and conventional white, red and/or black grapes were collected at regular intervals during the winter (when grapes were imported from South Africa) and summer (when grapes were imported from Mediterranean countries) in two consecutive years (2015 and 2016). All experimental factors significantly affected the grape composition and ANOVA also detected a wide range of significant interaction between factors (Table 2, Table 3 and Table 4). The effects of the different factors are described in separate sections below.

### 3.1. Effects of Production Region and Year on Grape Composition

Since only red and white grapes were available in UK supermarkets from both (a) organic and conventional production and (b) production regions (South Africa in winter and Mediterranean in summer) main effects of production region and interactions of production region with year, grape type and/or production systems could only be assessed for red and white varieties (Table 2).

For white and red grapes from both production regions and black grapes from the Mediterranean samples were collected in two successive years (2015 and 2016) and the year was included in the ANOVA to estimate effects of production year (and associated differences in climatic conditions during the grape growing season) on grape composition.

Significant main effects of production region were detected for five of the eight composition parameters assessed (Table 2). Total DM and sugar (BRIX) concentrations in pulp were found to be slightly (~3%), but significantly higher in samples from South Africa, while total phenolic concentrations (TPC) and antioxidant activity by TEAC (TAA_TEAC_), were significantly higher (by 14%, 18% and 22% respectively) in samples from the Mediterranean. The production region had no significant main effect on anthocyanin concentrations (Table 2).

Significant 2-way interactions between grape type and production region were detected for the TPC and TAA_DPPH_ (Table 3). When these interactions were further investigated, red grapes from the Mediterranean were found to have significantly higher TPC and TAA_DPPH_ than grapes from South Africa (27% and 26% respectively) while white grapes from the two regions had similar levels of TPC and TAA_DPPH_ (Table 3).

A significant main effect of year was only detected for TAA_TEAC_, which was significantly higher in 2015 than in 2016 (Table 2). However, there were also ranges of significant 2-, 3- and 4-way interactions between year and other factors (Table 2). When the 3-way interaction between year, production region and the grape type was further investigated, significant differences between regions were detected only for (a) the juice sugar content in red grapes in 2015, with higher levels detected in

Mediterranean grapes, (b) TAA_DPPH_ in both red and white grapes in 2015 and red grapes in 2016 with higher levels detected in Mediterranean grapes in both years and (c) TAA_TEAC_ in both white and red grapes in 2015, but only white grapes in 2016 with levels higher in Mediterranean grapes in 2015, but South African white grapes in 2016 (Table 4).

Significant 3-way and 4-way interactions with the production system (organic vs. conventional) were also detected in the 4-factor ANOVA for a range of composition parameters (Table 2) and are described in detail in Section 3.3 and Tables 8–10 (see below).

### 3.2. Effect of Grape Type (White vs. Red vs. Black) on Grape Composition

Black grapes from organic production were not available during the winter grape season (when grapes are imported from South African) main effects of all three grape types (black, red and white) and interactions with year and production systems could therefore only be assessed for grapes produced in Mediterranean countries during the summer grape season (Table 5).

Three-factor (year, production system, grape type) ANOVA detected significant main effects of grape type for all composition parameters assessed when data obtained from samples of three grape types (white, red and black) produced in the Mediterranean were analyzed (Table 5).

The DM, SC (pulp) and SC (juice) were significantly higher in red than in both white and black grapes, which had similar concentrations. The TPC, TAA_DPPH_ and TAA_TEAC_ were highest in black, intermediate in red and lowest in white grapes, but only the difference between black and white grapes was significant for all three parameters (Table 5). The largest differences between grape types were detected for the TAC, with black grapes found to have more than 5 times higher TAC than red grapes, while red grapes had a more than 8 times higher TAC than white grapes (Table 5).

Three-factor ANOVA also detected a significant main effect of year for TAA_TEAC_ (higher in 2015 than 2016) and TAC (higher in 2016 than 2015) and a range of significant 2-way interactions between (a) year and grape type (Table 5). Further examination of the interactions between year and grape type showed significant differences between years were detected for (a) the dry matter content and TAA_TEAC_ in red grapes only (higher in 2016) and (b) TAC in black grapes only (higher in 2016). (Table 6). In 2015 white and red grapes had similar TAA_TEAC_ levels which were both significantly lower than those found in black grapes, while in 2016 red grapes had significantly higher TAA_TEAC_ than white grapes, which were similar to those found in black grapes (Table 6).

Interactions between grape type, and production systems and/or year identified in the 3-factor ANOVA (Table 5) are described in detail in Section 3.3 (see below).

Since white grapes were found to have very low anthocyanin concentrations (Table 2 and Table 5), anthocyanin profiles (concentrations of individual anthocyanins) were only assessed and compared in red and black grapes (Table 7).

When anthocyanin profiles were compared, significant main effects were only detected for grape type, but not the year or production system. Concentrations of delphinidin 3-*O*-glucoside, penunidin 3-*O*-gluciside, malvidin 3-*O*-glucoside, peonidin 3-*O*-p-coumaroylglucoside and malvidin 3-*O*-p-coumaroylglucoside were significantly and substantially (between 8 and 31 times) higher in black than red grapes. In contrast, concentrations of cyanindin 3-*O*-glucoside and peonidin 3-*O*-glucocside were not significantly different in red and black grapes (Table 7).

Significant 2-way interactions between grape type and year were detected for delphinidin 3-*O*-glucoside, petunidin 3-*O*-gluciside, malvidin 3-*O*-glucoside and showed that the difference between red and black grapes was only significant in one of the two years in which samples were collected (individual data not shown).

Three-way Interactions between production system, year and grape type identified by 3-factor ANOVA (Table 7) are described in detail in Section 3.3 (see below).

### 3.3. Effect of Production System (Organic vs. Conventional) on Grape Composition

When data from white and red grapes collected in both the summer and winter seasons were compared significant main effects of the production system were detected for three of the eight nutritional composition parameters assessed (Table 2). The DM, TPC and TAA_TEAC_ were slightly, but significantly higher (by 3%, 16% and 24% respectively) in organic grapes, while the TAC was 44% lower in organic grapes (Table 2). Significant interactions between the production system and grape type were detected for TAC only (Table 2). The TAC was significantly higher (~50%) in conventional than organic red grapes, but at similar levels in organic and conventional white grapes (Table 8).

Significant 3-way interactions between production system, production region and grape type (red vs. white) were detected for (a) the sugar content in both pulp and juice, (b) TAA_TEAC_ and (c) TAC (Table 2). When the 3-way interaction was further investigated only white grapes produced in the Mediterranean were found to have significantly higher sugar contents and TAA_TEAC_ in organic compared to conventional samples (Table 9).

Significant 4-way interactions between production system, year, production region and grape types (red vs. white) were only detected for TAC (Table 10). When the 4-way interaction was further investigated no significant differences in TAC content in white grapes produced in different years, regions and production systems (Table 10). In contrast, the TAC in organic red grapes from South Africa in 2015 and from Mediterranean countries in both years was compared to their conventional comparators; while there was no significant difference in TAC between organic and conventional red grapes from South Africa in 2016 (Table 10).

When data for all three grape types produced in the Mediterranean were analyzed, a significant interaction between the production system and grape type was detected for total TAA_TEAC_ only (Table 5). Significantly higher TAA_TEAC_ in organic than conventional samples were only detected for white grapes, while production systems had no significant effect on TAA_TEAC_ in red and black grapes (Table 11) produced in Mediterranean countries.

Although no significant main effects of production system on individual anthocyanins were detected, 3-factor ANOVA identified 3-way interactions between production system, year and grape type (red vs. black) (Table 7). When these interactions were further investigated, contrasting effects of the production system were detected in different years (2015 and 2016) especially for black grapes (Table 12). In 2015 concentrations of delphinidin 3-*O*-glucoside, penunidin 3-*O*-gluciside, peonidin 3-*O*-glucoside were higher in organic, while in 2016 they were higher in conventional black grapes, but differences were only significant in 2016. In contrast, conventional red grapes had higher peonidin 3-*O*-glucoside concentrations than organic red grapes in both 2015 and 2016, but the difference was only significant in 2015 (Table 12).

### 3.4. Effects of Variety Choice on Grape Composition

Supplementary ANOVA was carried out to identify the effects of variety and interactions between variety and production system. The available data allowed separate 2-factor ANOVAs to be carried for (a) white grapes from South Africa (varieties EarlySweet, Prime, Sugraone and Thompson) collected in 2015, (b) red grapes from South Africa (varieties Allison, Crimson, Flame and Sweet Celebration) and the Mediterranean (varieties Allison, Crimson, Flame and Scarlotta) collected in 2015, (c) black grapes from the Mediterranean (varieties Autumn Royal and Midnight Beauty) collected in 2015 and (d) red grapes from the Mediterranean (varieties Allison and Crimson) collected in 2016. It should be pointed out that the number of samples available for this supplementary 2-factor ANOVA was low for many varieties (Tables S1–S9).

Significant main effects of variety were detected for the (a) dry matter and sugar content, TPC and TAA_DPPH_ of white grape produced in South Africa in 2015 (Table S1), (b) sugar content, TPC, TAA_DPPH_ and TAA_TEAC_ of red grapes produced in the Mediterranean and South Africa in 2015 (Tables S2 and S3) and (c) sugar content, TAA_DPPH_ and TAA_TEAC_ and TAC in black grapes produced in the Mediterranean in 2015 (Table S4). However, for red grapes from the Mediterranean collected in 2016 no significant main effects of variety were detected (Table S5).

Significant interactions between the production system and variety choice were also detected for a range of composition parameters in white, red and black grapes collected in 2015 (Table S1–S4). Further examination of these interactions showed that significant effects of production were limited to specific varieties. In white grapes from South Africa the TPC was significantly higher in organic grapes of the variety “Sugaraone”, but conventional grapes of the variety “Prime”, while no significant effect of production system was detected for the varieties “Early Sweet” and “Tompson” (Table S1). In red grapes from South Africa the pulp sugar content was significantly higher in conventional than organic grapes of the variety “Crimson”, while no significant effect of production system was detected for the varieties “Allison”, “Flame” and “Scarlotta” (Table S2). In black grapes produced in the Mediterranean the juice sugar content, and TAC was significantly higher in organic than conventional grapes of the variety Midnight Beauty, while no significant effect of production system was detected for the variety “Autumn Royal” (Table S4).

The range of grape varieties available in UK supermarkets during the winter (from South African production) and summer (from the Mediterranean) different considerably and for most varieties it was not possible to obtain samples from both organic and conventional production in both seasons. The season could therefore not be included as a factor in the supplementary ANOVA carried out to identify potential effects of variety except for red grapes where samples of two varieties (“Crimson” and “Flame”) were available from both production regions in 2015 (Table 6).

Three-factor ANOVA of data for the red varieties “Crimson” and “Flame” identified significant main effects of production region for all composition parameters. Specifically, grapes from the Mediterranean had a higher TPC, TAA and TAC content, while grapes from South Africa had a higher dry matter and sugar content (Table S6).

Significant interactions between variety, production region, and/or production system were also detected for most composition parameters studied (Table S6). Further examination of the two-way interactions showed that (a) the juice sugar content was higher in organic than conventional grapes from South Africa, while there was no significant effect of the production system in grapes produced in the Mediterranean (Table S6), (b) “Crimson” grapes produced in South Africa had a significantly higher pulp and juice sugar content than grapes of the same variety produced in the Mediterranean, while the was no significant effect of production region for the variety “Flame” (Table S6), (c) TAA_DPPH_ and TAA_TEAC_ were significantly higher in “Flame” than “Crimson” grapes produced in the Mediterranean, but higher in “Crimson” than “Flame” grapes produced in South Africa (Table S6), (d) TAC was significantly higher in conventional than organic grapes of the variety Crimson, while there was no significant effect of production system for the variety Flame (Table S6). Further examination of the three-way interaction between production region, grape variety and production system showed that a significantly higher juice sugar content in conventional compared to organic grapes could only be detected for red grapes of the variety “Crimson” produced in the Mediterranean (Table 6).

The data available allowed anthocyanin profiles to be compared in samples from (a) the red varieties Crimson and Flame from Mediterranean countries collected in 2015 (Table S7), (b) the red varieties Crimson and Allison from South Africa collected in 2015 (Table S8) and (c) the black varieties Autumn royal and Midnight Beauty from Mediterranean countries in 2015 (Table S9).

When two red variety produced in the Mediterranean were compared the variety Flame had substantially (more than 15 times) higher delphinidin 3-*O*-glucoside, cyanindin 3-*O*-glucoside and petunidin 3-*O*-glucoside concentrations compared to the variety Crimson, while no significant main effect of variety could be detected for the other four anthocyanins assessed (Table S7). However, there were also significant interactions between variety and production system for six of the seven anthocyanins monitored (Table S7). When these interactions were further investigated significantly higher concentrations of peonidin 3-*O*-glucoside, peonidin 3-*O*-p-coumaroyl and malvidin 3-*O*-p-coumaroyl were detected in conventional compared to organic grapes of the variety Crimpson. In contrast, organic grapes of the variety Flame had higher concentrations of delphinidin 3-*O*-glucoside, petunidin 3-*O*-glucoside, malvidin 3-*O*-glucoside and malvidin 3-*O*-glucoside than their conventional comparators (Table S7).

When two red varieties produced in South Africa were compared, no significant main effects of variety were detected, but significant interactions between variety and production system were found for four of the seven anthocyanins monitored (Table S8). When these interactions were further investigated, organic grapes of the variety Allison were found to have significantly lower concentrations of cyanindin 3-*O*-glucoside and peonidin 3-*O*-glucoside, but significantly higher peonidin 3-*O*-p-coumaroyl glucoside and malvidin 3-*O*-p-coumaroyl glucoside concentrations than conventional Allison grapes (Table S8). In contrast, no significant differences were detected between organic and conventional grapes of the variety Allison (Table S8).

When two black varieties produced in South Africa were compared, no significant main effects of variety were detected and a significant interaction between variety and production system was only found for peonidin 3-*O*-glucoside (Table S9). When the interactions were further investigated, peonidin 3-*O*-glucoside concentrations were significantly higher in organic compared to conventional grapes of the variety Midnight Beauty (Table S9). In contrast, the production system had no significant effect of the production system was detected in the variety Autumn royal and peonidin 3-*O*-glucoside concentrations were numerically higher in conventional grape samples (Table S9).

## 4. Discussion

The UK retail survey described here was conducted to investigate the effect of production season/supply chain (winter vs. summer), grape type (white vs. red vs. black) and production systems (organic vs. conventional) on the nutritional composition of table grape products available to consumers in UK supermarkets. Retail surveys are the most accurate approach to assess differences in composition/quality between different food products at the point of purchase by consumers (14,59).

However, one of the main limitations of retail surveys is that information on the exact production protocols and pedo-climatic conditions on the farms supplying supermarkets is usually not available (14,59). In the study reported here it was, therefore, possible to identify effects of specific agronomic/management protocols (e.g., fertilization, crop protection, tillage, irrigation, ripening stage at harvest), environmental drivers and post-harvest conditions (e.g., storage, cooling, packaging) on the grape composition parameters assessed in this study. Results are therefore discussed in the context of existing published information on the effects of pedoclimatic, agronomic and supply chain-related parameters on phenolic and anthocyanin concentrations, and antioxidant activity in table grapes.

### 4.1. Effect of Year and Production Region on the Nutritional Composition of Table Grapes

Effects of contrasting climatic conditions between years on grape quality are well documented [16,17,18,19,20,21,22,23,24,25,26,27,28], but this is to our knowledge the first study in which the composition of grapes from the two main production regions (Mediterranean vs. South Africa) that supply the UK and other European countries were investigated. The lower TPC and TAA found in grapes sampled during the winter season (when grapes were from South Africa) may have been due to a range of factors including differences in pedoclimatic conditions and agronomic/cultural practices used in the Mediterranean and South African grape production areas, including transport distance/time [45,46,47].

Winter season grapes produced in the southern hemisphere spend longer (15–40 days) in transport than summer season grapes (2–5 days) produced in the Mediterranean [45]. They may also be harvested at a slightly earlier ripening stage, to compensate for longer transport times [47]. Both sugar and total phenolic concentrations (and in red/black grapes anthocyanin) are known to increase until physiological ripeness of grapes [35,48,49,50]. However, grape berries are non-climacteric fruit and postharvest, the sugar content of grapes remains very stable under commercial cold storage conditions [45,51]. This allows sugar content to be used as a marker for the ripening stage at harvest. The finding of similar sugar contents (BRIX) in grapes from both production regions in this study, therefore, suggests that there were no substantial differences in ripening stage at harvest between grapes imported from the Mediterranean and South Africa. In addition, there is evidence that the antioxidant activity of grapes and other fruit and vegetables, remains relatively stable during cold storage until they visibly spoil [52].

Differences in the (a) postharvest treatment (e.g., use of sulfur dioxide, heat, antimicrobial and ozone treatments or anti-browning agents) protocols [53] and (b) range of varieties sources produced or sourced from South Africa and the Mediterranean may also have contributed to the observed effects of production region. The latter is supported by the findings of this study that (a) the range of red and white varieties available in the winter (when grapes were from South Africa) and summer season (when grapes were from Mediterranean countries) was different and (b) there are significant differences in sugar content, phenolic concentrations and/or antioxidant activity (TEAC) between varieties of the same grape type.

### 4.2. Effect of Grape Type and Variety on the Nutritional Composition of Table Grapes

Both PCA and ANOVA clearly identified grape type as the main explanatory variable/driver for differences in nutritionally desirable total phenolic and anthocyanin concentrations and antioxidant activity in grapes. In contrast year, production season and production system explained less of the variation in the nutritional composition of table grapes at the point of retail.

Our findings that (a) total phenolic and anthocyanin concentrations and antioxidant activity were highest in black, intermediate in red and lowest in white red grapes and (b) magnitude of difference in anthocyanin concentrations (five-times higher in black than red and more than seven times higher in red than white grapes) is consistent with the results of previous studies [54,55,56].

The profile of grape varieties produced is known to differ between countries/regions [2,46] and results from this survey suggest that there are also differences in the range of varieties available as organic and conventional products in the UK market. This is consistent with previous studies in other crops (e.g., cereals, potato) that reported (a) differences in variety choice between organic and conventional production and/or (b) that organic farmers tend to select more resistant, robust and/or traditional varieties [57,58]. For cereals, there is evidence from long-term field experiments that differences in variety choice between organic and conventional systems partially explain the significantly higher phenolic and mineral micronutrient concentrations found in organic compared to conventional wheat flour in recent retail surveys in the UK and Germany [58,59].

Supplementary analysis of composition differences between varieties of the same grape type identified in this study suggests that differences in the profile of varieties available as organic and conventional table grapes may also have contributed to overall effects of production systems detected (higher TPC, TAA_TEAC_ in organic and higher TAC in conventional table grapes) in this study.

The finding that there were no significant differences in phenolic and/or antioxidant levels between organic and conventional grapes for the majority of varieties, while significantly higher levels were found in grapes from organic or conventional production for some varieties is consistent with the overall trend of previous studies [16,18,19,20,21,22,60].

### 4.3. Effect of Production System on the Nutritional Composition of Table Grapes

The finding of overall higher total phenolic concentrations and antioxidant activity in organic than conventional white and red table grapes in this study is consistent with the results of a recent meta-analysis of comparative (organic vs. conventional) crop composition data which reported 20% higher antioxidant activity in organic crops [14]. However, in the meta-analysis data from different crops or crop groups were pooled, and it, therefore, remained unclear for which individual crop species, organic management practices result in significant differences in antioxidant levels.

Overall higher (~15%) TAA_TEAC_ in organic than conventional white and red grapes was also reported in a recent farm survey-based study in Crete, which compared the effect of organic and conventional production system on the nutritional composition of traditional local grape varieties (2 white and 1 red) used as table grapes and for wine-making [60].

However, this study also demonstrated that the effect of the production system differs between varieties for a range of composition parameters assessed (see Section 4.2 above). For white grapes, the interactions between variety and production system were similar to that reported in a recent farm survey-based study in Crete. The study found significantly higher (~50%) antioxidant activity (TEAC) in organic compared to conventional white grapes but the effect of the production system was only significant for one of the two white varieties included in the survey [60].

This study also suggests that the effect of the production system can be confounded by both (a) production year or season (and associated pedo-climatic and supply chain differences) and (b) grape type and/or variety. The confounding effects of year/season (and associated pedoclimatic differences) and grape type/varieties identified in this study, may also explain the contrasting results of previous studies that compared antioxidant activity and/or concentrations in grapes from organic and conventional grapes. For example, in two studies carried out in Brazil, Toaldo, et al. [37] reported higher TAA_TEAC_ and TAA_DPPH_ in red grapes, while da Silva Haas, et al. [38] found no significant effect of production system on TAA in red grapes. In two studies carried out in Italy, Tassoni, et al. [22,23] reported TAA_DPPH_ is higher in organic than conventional red Lambrusco grapes, but higher in conventional white Albana and red Sangioves grapes, while no effect of production system was found in white Pignoletto grapes.

These interactions should be considered in the design of future retail and farm surveys, and experimental studies.

### 4.4. Potential Nutritional/Health Impacts of Consuming Different Table Grape Products

There is increasing epidemiological evidence that the antioxidant/(poly)phenol compounds in food crops have protective effects against a range of chronic diseases [18,60,61,62]. Grape types/varieties, grape production systems and supply chains that deliver higher TPC, TAA and/or TAC concentrations would therefore be expected to provide “added nutritional value” since they allow an increase in dietary antioxidant intake without a simultaneous increase in energy intake. However, there is still considerable uncertainty on whether there are linear dose-response relationships between dietary intake of plant phenolics/antioxidants and their physiological effects in humans. This is mainly because there is still relatively limited information about the rates of uptake, bioavailability/metabolism and physiological effects of different phenolics/antioxidants in the gut environment, and their mode(s) of action (e.g., as an antioxidant, anti-inflammatory and/or signaling molecules) following uptake into the body [18,62].

However, this study has identified changes in the grape type and to a lesser extent variety choice as the most important strategy to increase dietary intakes of phenolic compounds and the antioxidant activity associated with them. Specifically, the study suggests that switching from white to red and especially black grape consumption will increase TPC, TAA and TAC intakes (and associated potential health benefits) at the same level of consumption, as reported previously [18,19,22,23].

The relatively small differences in TPC and TAA levels between grapes from the two production regions are unlikely to be nutritionallyrelevant. However, results suggest that targeted changes to variety choice and/or supply chain-related parameters may allow TPC and TAA levels and associated potential health benefits to be further increased in grapes imported from South Africa. However, this would require further studies to identify the parameters responsible for composition differences between seasons, which may include (a) variety choice, (b) primary production and harvest protocols, (c) pedo-climatic conditions and/or (d) postharvest treatments, storage and transport conditions.

This study does not allow the potential overall nutritional/health impact of switching to organic grape consumption to be assessed. This is mainly, because organic and conventional crops were previously shown to not only differ in antioxidant activity and phenolic content (which were assessed in this study), but also concentrations of other nutritionally-relevant compounds (including essential micro-nutrients, toxic cadmium and pesticide residue) [14,17,63] which were not assessed in the study reported here.

## Figures and Tables

**Table 1:** Varieties and number of samples of organic and conventional white, red and black grapes collected and analyzed in 2015 and 2016.

Season Grape Type	Varieties Available (No. of Samples)	
	Organic	Conventional
**Winter 2015**		
White	Early Sweet (*n* = 2)	Early Sweet (*n* = 4)
	Prime (*n* = 7)	Prime (*n* = 8)
	Sugraone (*n* = 11)	Sugraone (*n* = 11)
	Sweet Sunshine (*n* = 2)	Sweet Sunshine (*n* = 1)
	Thompson (*n* = 7)	Thompson (*n* = 21)
	Muscat (*n* = 1)	
	Regal (*n* = 12)	
	Sundance (*n* = 4)	
Red	Allison (*n* = 6)	Allison (*n* = 2)
	Crimson (*n* = 6)	Crimson (*n* = 19)
	Flame (*n* = 4)	Flame (*n* = 6)
	Sweet Celebration (*n* = 12)	Sweet Celebration (*n* = 2)
	Jack’s Sallute (*n* = 8)	Ralli (*n* = 4)
	Sunred (*n* = 2)	Scarlotta (*n* = 4)
		Timco (n = 1)
Black	Autumn Royal (*n* = 3)	Autumn Royal (*n* = 5)
	IFG16 (*n* = 5)	Desert (*n* = 1)
	IFG17 (*n* = 1)	
	Sweet Surrender (*n* = 3)	
**Summer 2015**		
White	Sugraone (*n* = 14)	Sugraone (*n* = 13))
	Superior (*n* = 3)	Superior (*n* = 1)
	Timpson (*n* = 3)	Timpson (*n* = 4)
	Regal (*n* = 13)	Mellisa (*n* = 1)
		Princess (*n* = 1)
		Thompson (*n* = 13)
Red	Allison (*n* = 2)	Allison (*n* = 2)
	Crimson (*n* = 13)	Crimson (*n* = 20)
	Flame (*n* = 5)	Flame (*n* = 5)
	Scarlotta (*n* = 10)	Scarlotta (*n* = 3)
Black	Autumn Royal (*n* = 4)	Autumn Royal (*n* = 4)
	Midnight Beauty (5)	Midnight Beauty (*n* = 7)
	Summer Royal (*n* = 4)	Summer Royal (*n* = 1)
		Melody (*n* = 1)
**Winter 2016**		
White	Thompson (*n* = 9)	Thompson (*n* = 10)
	Early Sweet (*n* = 1)	Sugraone (*n* = 3)
	Regal (*n* = 1)	
	Sundance (*n* = 5)	
	Sweet Globe (*n* = 3)	
	Sweet Sunshine (*n* = 1)	
Red	Flame (*n* = 1)	Flame (*n* = 1)
	Jack’s Sallute (*n* = 2)	Crimson (*n* = 8)
	Sweet Celebration (*n* = 8)	Ralli (*n* = 1)
Black	Autumn Royal (*n* = 1)	
**Summer 2016**		
White	Sugraone (*n* = 11)	Sugraone (*n* = 9)
	Superior (*n* = 2)	Superior (*n* = 2)
	Thompson (*n* = 1)	Thompson (*n* = 5)
	Timpson (*n* = 4)	Timpson (*n* = 2)
	Sophia (*n* = 3)	Cotton Candy (*n* = 1)
	Sublima (*n* = 3)	Early Sweet (*n* = 1)
		ITUM (*n* = 2)
Red	Allison (*n* = 3)	Allison (*n* = 2)
		Scarlotta (*n* = 1)
	Crimson (*n* = 4)	Crimson (*n* = 5)
	Sweet Celebration (*n* = 2)	Sweet Celebration (*n* = 1)
Black	Midnight Beauty (*n* = 6)	Midnight Beauty (*n* = 6)
	Summer Royal (*n* = 6)	Summer Royal (*n* = 1)
	Vitroblack (*n* = 3)	Autumn Royal (*n* = 5)
		Melody (*n* = 2)

**Table 2:** Effect of, and interactions between, year, production system (organic (ORG) vs. conventional (CON), production region (South Africa (RSA) vs. the Mediterranean (MED)) and table grape type (red vs. white) on the dry matter content (DM), sugar content (SC) of pulp/juice, total phenolic content (TPC), total antioxidant activity (TAA; DPPH and ABTS assays) and total anthocyanin content (TAC) in grape samples from a UK supermarket survey (4-factor ANOVA, the values presented are means ± SE).

Factors	Dry Matter Content %	Sugar Content (SC)		Total Phenolic Content (TPC) mg GAE kg^−1^	Antioxidant Activity (TAA)		Total Anthocyanin Content (TAC)	
		(Pulp) Brix°	(Juice) Brix°		DPPH µmol TE g^−1^	TEAC µmol TE g^−1^	mg cyan kg^−1^	mg mal kg^−1^
**Year (Yr)**								
2015 (*n* = 292)	19.5 ± 0.1	17.6 ± 0.1	17.8 ± 0.1	1583 ± 36	78 ± 2	7.7 ± 0.3	48 ± 4	51 ± 4
2016 (*n* = 118)	19.7 ± 0.3	17.7 ± 0.2	17.9 ± 0.2	1551 ± 44	77 ± 3	5.1 ± 0.4	45 ± 6	48 ± 7
**Production system (PS)**								
ORG (*n* = 210)	19.8 ± 0.2	17.7 ± 0.1	17.9 ± 0.2	1689 ± 42	78 ± 2	7.7 ± 0.4	39 ± 3	41 ± 3
CON (*n* = 200)	19.3 ± 0.2	17.6 ± 0.1	17.8 ± 0.1	1452 ± 37	77 ± 2	6.2 ± 0.3	56 ± 6	59 ± 6
**Production region (PR)**								
RSA (*n* = 220)	19.8 ± 0.2	17.9 ± 0.1	18.0 ± 0.1	1481 ± 32	72 ± 2	6.3 ± 0.3	48 ± 4	51 ± 5
MED. (*n* = 190)	19.3 ± 0.2	17.3 ± 0.2	17.7 ± 0.2	1681 ± 49	85 ± 3	7.7 ± 0.4	46 ± 5	49 ± 5
**Grape type (GT)**								
red (*n* = 174)	20.6 ± 0.2	18.3 ± 0.1	18.5 ± 0.1	1827 ± 42	104 ± 2	7.1 ± 0.4	98 ± 6	103 ± 6
white (*n* = 236)	18.8 ± 0.2	17.2 ± 0.1	17.3 ± 0.1	1386 ± 34	59 ± 1	6.9 ± 0.4	10 ± 1	11 ± 1
**ANOVA** *p*-values								
**Main effects**								
Yr	NS	NS	NS	NS	NS	**<0.0001**	NS	NS
PS	**0.0179**	NS	NS	**<0.0001**	NS	**0.0008**	**0.0001**	**0.0001**
PR	**0.0270**	**0.0014**	NS	**0.0002**	**<0.0001**	**0.0002**	NS	NS
GT	**<0.0001**	**<0.0001**	**<0.0001**	**<0.0001**	**<0.0001**	**NS**	**<0.0001**	**<0.0001**
Interactions *								
Yr:PS	NS	NS	*0.0797*	**0.0005**	NS	**0.0363**	NS	NS
Yr:PR	NS	NS	NS	**0.0327**	NS	**<0.0001**	**0.0244**	**0.0237**
Yr:GT	**0.0453**	NS	NS	**0.0306**	**<0.0001**	NS	NS	NS
PS:PR	NS	**0.0233**	**0.0305**	NS	NS	NS	NS	NS
GT:PS	NS	NS	NS	NS	NS	NS	**<0.0001 ^3^**	**<0.0001 ^3^**
GT:PR	NS	NS	NS	**0.0004 ^1^**	**<0.0001 ^1^**	NS	NS	NS
Yr:PR:GT	*0.0858*	NS	**0.0112 ^2^**	NS	**0.0155 ^2^**	**<0.0001 ^2^**	**0.0003**	**0.0004**
PS:PR:GT	NS	**0.0300 ^4^**	**0.0204 ^4^**	NS	NS	**0.0036 ^4^**	**0.0438**	**0.0434**
Yr:PS:PR:GT	NS	NS	NS	NS	NS	NS	**0.0187 ^5^**	**0.0188 ^5^**

GAE, Gallic acid equivalent; TE, Trolox equivalent; cyan, cyanidin 3-glucoside equivalent; mal, malvidin 3-glucoside equivalent; *p*-values in *italic* are for trends (0.1 < *p* < 0.05); *p*-values in **bold** are significant (*p* < 0.05). * only interactions for which significant results were detected are shown; **^1^** see Table 3 for interaction means ± SE; **^2^** see Table 4 for interaction means ± SE; **^3^** see Table 8 for interaction means ± SE; **^4^** see Table 4 for interaction means ± SE; **^5^** see Table 10 for interaction means ± SE.

**Table 3:** Interactions means ± SE for the effects of grape type and production region on the total phenolic content and antioxidant activity in table grapes.

Parameter	Factor 1	Factor 2	
		Production Region	
	Grape Type	South Africa	Mediterranean
Total phenolic content (mg GAE kg^−1^)	White	1365 ± 44 **a B**	1409 ± 52 **a B**
	Red	1629 ± 41 **b A**	2070 ± 72 **a A**
Antioxidant activity (DPPH, µmol TE g^−1^)	White	55 ± 1 **a B**	62 ± 2 **a B**
	Red	93 ± 2 **b A**	117 ± 4 **a A**

GAE, Gallic acid equivalent; TE, Trolox equivalent; For each parameter assessed means labeled with the same lower case letter within the same row and same capital letters within the same column are not significantly different (General Linear Hypothesis test *p* < 0.05).

**Table 4:** Interactions means ± SE for the effects of year, production region and grape type on the total sugar content and antioxidant activity (TEAC) and TAC in table grapes.

Parameter			Factor 3	
	Factor 1	Factor 2	Grape Type	
	Year	Production Region	White	Red
Sugar content (juice) Brix°	2015	South Africa	17.2 ± 0.2 **b A**	18.8 ± 0.2 **a A**
		Mediterranean	17.5 ± 0.3 **a A**	17.9 ± 0.2 **a B**
	2016	South Africa	17.6 ± 0.3 **b A**	18.7 ± 0.4 **a AB**
		Mediterranean	17.1 ± 0.4 **b A**	19.3 ± 0.4 **a A**
Antioxidant activity (DPPH) (µmol TE g^−1^)	2015	South Africa	56.7 ± 0.8 **b B**	91.4 ± 2.6 **a B**
		Mediterranean	63.5 ± 2.2 **b A**	110.0 ± 4.3 **a A**
	2016	South Africa	51.6 ± 2.2 **b B**	98.7 ± 2.3 **a B**
		Mediterranean	60.1 ± 3.5 b **A B**	140.0 ± 1.2 **a A**
Antioxidant activity (TEAC) (µmol TE g^−1^)	2015	South Africa	5.9 ± 0.4 **a B**	6.4 ± 0.5 **a B**
		Mediterranean	10.1 ± 0.8 **a A**	9.4 ± 0.7 **a A**
	2016	South Africa	8.8 ± 1.3 **a A**	3.8 ± 0.3 **b C**
		Mediterranean	2.8 ± 0.2 **b C**	5.8 ± 0.3 **a B C**

TE, Trolox equivalent; cyan, cyanidin 3-glucoside equivalents; mal, malvidin 3-glucoside equivalents; For each parameter, assessed means labeled with the same lower case letter within the same row and capital letters within the same column are not significantly different (General Linear Hypothesis test *p* < 0.05).

**Table 5:** Effect of, and interaction between, year, production system (ORG vs. CON), and table grape type (black vs. red vs. white) on the DMcontent, SC of pulp/juice, TPC, total antioxidant activity (TAA; DPPH and ABTS assays) and TAC in grape samples produced in the Mediterranean (3-factor ANOVA, the values presented are means ± SE).

Factors	Dry Matter Content %	Sugar Content (SC)		Total Phenolic Content	Antioxidant Activity (TAA)		Total Anthocyanin Content (TAC)	
		(Pulp) Brix°	(Juice) Brix°	(TPC) mg GAE kg^−1^	DPPH µmol TE g^−1^	TEAC µmol TE g^−1^	mg cyan kg^−1^	mg mal kg^−1^
**Year (Yr)**								
2015 (*n* = 152)	19.1 ± 0.2	17.1 ± 0.2	17.6 ± 0.2	1858 ± 63	93 ± 3	11.3 ± 0.6	116 ± 17	123 ± 18
2016 (*n* = 93)	19.2 ± 0.3	17.4 ± 0.3	17.7 ± 0.3	1735 ± 60	98 ± 4	4.3 ± 0.2	234 ± 33	247 ± 35
**Production system (PS)**								
ORG (*n* = 124)	19.4 ± 0.3	17.4 ± 0.2	17.9 ± 0.2	1891 ± 67	96 ± 4	9.5 ± 0.7	158 ± 25	167 ± 27
CON (*n* = 121)	18.8 ± 0.2	17.0 ± 0.2	17.3 ± 0.2	1730 ± 62	94 ± 4	7.8 ± 0.5	164 ± 22	173 ± 23
**Grape Type (GT)**								
black (*n* = 55)	18.5 ± 0.3 **b**	16.8 ± 0.3 **b**	17.3 ± 0.3 **b**	2262 ± 90 **a**	130 ± 3 **a**	11.9 ± 1.2 **a**	557 ± 41 **a**	588 ± 43 **a**
red (*n* = 78)	20.2 ± 0.2 **a**	17.9 ± 0.2 **a**	18.2 ± 0.2 **a**	2070 ± 72 **a**	117 ± 4 **b**	8.6 ± 0.6 **b**	95 ± 10 **b**	100 ± 11 **b**
white (*n* = 112)	18.7 ± 0.3 **b**	17.0 ± 0.2 **b**	17.3 ± 0.2 **b**	1410 ± 52 **b**	62 ± 2 **c**	7.1 ± 0.6 **b**	13 ± 1 **c**	13 ± 1 **c**
**ANOVA**								
***p*-values**								
**Main effects**								
Yr	NS	NS	NS	NS	NS	**<0.0001**	**<0.0001**	**<0.0001**
PS	*0.0875*	*0.0837*	**0.0253**	**0.0294**	NS	**0.007**	NS	NS
GT	**0.0001**	**0.0014**	**0.0047**	**<0.0001**	**<0.0001**	**<0.0001**	**<0.0001**	**<0.0001**
Interactions *								
Yr:PS	NS	NS	NS	**0.0005**	NS	**0.0109**	NS	NS
Yr:GT	**0.0456 ^1^**	NS	NS	NS	**0.0003 ^1^**	**<0.0001 ^1^**	**0.0013 ^1^**	**0.0013 ^1^**
PS:GT	NS	NS	NS	NS	NS	**0.0247 ^2^**	NS	NS

GAE, Gallic acid equivalent; TE, Trolox equivalent; cyan, cyanidin 3-glucoside equivalent; mal, malvidin 3-glucoside equivalent; means labeled with the same lower case letter within the same column are not significantly different (General Linear Hypothesis test *p* < 0.05); *p*-values in *italic* are for trends (0.1 < *p* < 0.05); *p*-values in **bold** are significant (*p* < 0.05). * only interactions for which significant results were detected are shown; **^1^** see Table 6 for interaction means ± SE; **^2^** see Table 11 for interaction means ± SE.

**Table 6:** Interactions means ± SE for the effects of grape type and year on the dry matter and juice sugar content, antioxidant activity (DPPH and TEAC) and anthocyanin content and in table grapes.

Parameter	Factor 1	Factor 2	
		Year	
	Grape Type	2015	2016
Dry matter content (%)	White	18.7 ± 0.3 **a A**	18.6 ± 0.5 **a B**
	Red	19.8 ± 0.2 **b A**	21.6 ± 0.6 **a A**
	Black	18.6 ± 0.5 **a A**	18.4 ± 0.5 **a B**
Antioxidant activity (DPPH, µmol TE g^−1^)	White	127 ± 6.5 **a A**	60 ± 3.5 **a B**
	Red	110 ± 4.3 **b B**	140 ± 1.2 **a A**
	Black	63 ± 2.2 **a C**	132 ± 2.5 **a A**
Antioxidant activity (TEAC, µmol TE g^−1^)	White	10.1 ± 0.8 **a B**	2.8 ± 0.2 **b B**
	Red	9.4 ± 0.7 **a B**	5.8 ± 0.3 **b A**
	Black	18.6 ± 1.7 **a A**	5.9 ± 0.5 **b A**
Anthocyanin content (mg cyan kg^−1^)	White	12 ± 1 **a C**	15 ± 1 **a C**
	Red	81 ± 10 **a B**	139 ± 27 **a B**
	Black	462 ± 61 **b A**	642 ± 50 **a A**
Anthocyanin content (mg mal kg^−1^)	White	12 ± 1 **a C**	16 ± 1 **a C**
	Red	86 ± 10 **a B**	147 ± 28 **a B**
	Black	487 ± 65 **b A**	677 ± 52 **a A**

TE, Trolox equivalent; cyan, cyanidin 3-glucoside equivalent; mal, malvidin 3-glucoside equivalent; For each parameter assessed means labeled with the same lower case letter within the same row and same capital letters within the same column are not significantly different (General Linear Hypothesis test *p* < 0.05).

**Table 7:** Effect of, and interaction between, year, production system (ORG vs. CON), and table grape type (black vs. red) on the content of individual anthocyanins in red and black grape samples from a UK supermarket survey (3-factor ANOVA, the values presented are means ± SE).

Factor	Concentrations of Individual Anthocyanins (mg FW kg^−1^)						
	Delphindin 3-*O*-Glucoside	Cyanindin 3-*O*-Glucoside	Petunidin 3-*O*-Glucoside	Peonidin 3-*O*-Glucoside	Malvidin 3-*O*-Glucoside	Peonidin 3-*O*-p-Coumaroyl Glucoside	Malvidin 3-*O*-p-Coumaroyl Glucoside
**Year** **(Yr)**							
2015 (*n* = 37)	29 ± 7	23 ± 5	51 ± 12	241 ± 39	567 ± 113	31 ± 6	264 ± 65
2016 (*n* = 15)	40 ± 22	24 ± 10	75 ± 37	273 ± 73	759 ± 321	37 ± 14	272 ± 126
**Production system (PS)**							
ORG (*n* = 26)	37 ± 11	21 ± 6	65 ± 18	207 ± 36	673 ± 183	34 ± 8	270 ± 82
CON (*n* = 26)	28 ± 13	25 ± 7	52 ± 20	294 ± 59	572 ± 162	32 ± 9	262 ± 84
**Grape type (GT)**							
black (*n* = 22)	67 ± 17	21 ± 8	128 ± 25	279 ± 64	1415 ± 181	67 ± 10	627 ± 93
red (*n* = 30)	7 ± 3	25 ± 5	7 ± 2	229 ± 39	41 ± 7	8 ± 2	2 ± 0.4
**ANOVA** ***p*-values**							
**Main effects**							
Yr	NS	NS	NS	NS	NS	NS	NS
PS	NS	NS	NS	NS	NS	NS	NS
GT	**<0.0001**	NS	**<0.0001**	NS	**<0.0001**	**<0.0001**	**<0.0001**
Interactions *							
Yr:PS	**0.0360**	NS	*0.0846*	NS	NS	NS	NS
Yr:GT	**0.0088**	NS	**0.0030**	NS	**0.0010**	NS	NS
Yr:PS:GT	**0.0110^1^**	NS	**0.0238 ^1^**	**0.0142 ^1^**	NS	NS	NS

*p*-values in *italic* are for trends (0.1 < *p* < 0.05); *p*-values in **bold** are significant (*p* < 0.05). * only interactions for which significant results were detected are shown; ^1^ see Table 12 for interaction means ± SE.

**Table 8:** Interactions means ± SE for the effects of grape type and production system on TAC in table grapes.

	Factor 1	Factor 2	
		Production System	
Parameter	Grape Type	Organic	Conventional
TAC (mg cyan kg^−1^)	White	11 ± 1 **a B**	8 ± 1 **a B**
	Red	77 ± 5 **b A**	118 ± 20 **a A**
TAC (mg mal kg^−1^)	White	12 ± 1 **a B**	9 ± 1 **a B**
	Red	82 ± 5 **b A**	124 ± 10 **a A**

cyan, cyanidin 3-glucoside equivalent; mal, malvidin 3-glucoside equivalent; For each parameter assessed means labeled with the same lower case letter within the same row and same capital letters within the same column are not significantly different (General Linear Hypothesis test *p* < 0.05).

**Table 9:** Interactions means ± SE for the effects of the production system, production region and grape type on TEAC and TAC in table grapes.

Parameter			Factor 3	
	Factor 1	Factor 2	Grape Type	
	Production Region	Production System	White	Red
Sugar content (pulp) Brix°	South Africa	Organic	17.0 ± 0.2 **b A B**	19.0 ± 0.2 **a A**
		Conventional	17.7 ± 0.2 **b A**	18.7 ± 0.2 **a A**
	Mediterranean	Organic	17.3 ± 0.4 **a A**	18.0 ± 0.3 **a B**
		Conventional	16.6 ± 0.3 **b B**	17.8 ± 0.3 **a B**
Sugar content (juice) Brix°	South Africa	Organic	17.1 ± 0.2 **b AB**	18.9 ± 0.2 **a A**
		Conventional	17.6 ± 0.2 **b AB**	18.6 ± 0.2 **a A**
	Mediterranean	Organic	17.8 ± 0.4 **a A**	18.3 ± 0.3 **a A**
		Conventional	16.9 ± 0.3 **b B**	18.2 ± 0.3 **a A**
Antioxidant activity (TEAC) (µmol TE g^−1^)	South Africa	Organic	7.0 ± 0.7 **a AB**	6.8 ± 0.7 **a AB**
		Conventional	6.2 ± 0.6 **a B**	4.8 ± 0.5 **a B**
	Mediterranean	Organic	8.6 ± 1.0 **a A**	8.5 ± 0.8 **a A**
		Conventional	5.6 ± 0.5 **b B**	8.7 ± 0.9 **a A**

TE, Trolox equivalent; cya, cyanidin 3-glucoside equivalents; mal, malvidin 3-glucoside equivalents; For each parameter assessed means labeled with the same lower case letter within the same row and same capital letters within the same column are not significantly different (General Linear Hypothesis test *p* < 0.05).

**Table 10:** Interactions means ± SE for the effects of year, production region, production systems and grape type on TAC in table grapes.

Parameter				Factor 4	
	Factor 1	Factor 2	Factor 3	Grape Type	
	Year	Production Region	Production System	White	Red
TAC mg cyan kg^−1^	2015	South Africa	Organic	6 ± 2 **b A**	89 ± 8 **a C**
			Conventional	5 ± 2 **b A**	119 ±12 **a B**
		Mediterranean	Organic	13 ± 1 **b A**	62 ± 7 **a D**
			Conventional	9 ± 2 **b A**	101 ± 17 **a C**
	2016	South Africa	Organic	14 ± 3 **b A**	78 ± 15 **a C D**
			Conventional	10 ± 2 **b A**	98 ± 18 **a C**
		Mediterranean	Organic	16 ± 2 **b A**	78 ± 7 **a C D**
			Conventional	13 ± 3 **b A**	201 ± 45 **a A**
TAC mg mal kg^−1^	2015	South Africa	Organic	7 ± 2 **b A**	94 ± 9 **a C**
			Conventional	6 ± 2 **b A**	126 ± 13 **a B**
		Mediterranean	Organic	14 ± 1 **b A**	66 ± 8 **a D**
			Conventional	10 ± 2 **b A**	106 ± 18 **a C**
	2016	South Africa	Organic	15 ± 3 **b A**	82 ± 16 **a C D**
			Conventional	10 ± 2 **b A**	97 ± 19 **a C**
		Mediterranean	Organic	17 ± 2 **b A**	82 ± 7 **a C D**
			Conventional	14 ± 3 **b A**	213 ± 47 **a A**

cyan, cyanidin 3-glucoside equivalents; mal, malvidin 3-glucoside equivalents; For each parameter assessed means labeled with the same lower case letter within the same row and same capital letters within the same column are not significantly different (General Linear Hypothesis test *p* < 0.05.

**Table 11:** Interactions means ± SE for the effects of grape type and production system on TEAC in table grapes produced in the Mediterranean.

Parameter	Factor 1	Factor 2	
		Production System	
	Grape Type	Organic	Conventional
Antioxidant activity (TEAC, µmol TE g^−1^)	White	8.6 ± 1.0 **a B**	5.6 ± 0.5 **b B**
	Red	8.5 ± 0.8 **a B**	8.7 ± 0.9 **a A**
	Black	12.6 ± 2.0 **a A**	11.1 ± 1.3 **a A**

TE, Trolox equivalent; For each parameter assessed means labeled with the same lower case letter within the same row and same capital letters within the same column are not significantly different (General Linear Hypothesis test *p* < 0.05).

**Table 12:** Interactions means ± SE for the effects of year, production system and grape type (red vs. black) on the concentrations of individual anthocyanin compounds in grapes produced in the Mediterranean.

Parameter			Factor 3	
	Factor 1	Factor 2	Grape Type	
	Year	Production System	Black	Red
delphinidin 3-*O*-glucoside (mg FW kg^−1^)	2015	Organic	74 ± 27 **a B**	15.7 ± 7.0 **b A**
		Conventional	32 ± 8 **a B**	5.3 ± 2.0 **b A**
	2016	Organic	69 ± 25 **a B**	0.2 ± 0.1 **b A**
		Conventional	193 ± 136 **a A**	0.8 ± 0.6 **b A**
petunidin 3-*O*-glucoside (mg FW kg^−1^)	2015	Organic	132 ± 392 **a B C**	12.6 ± 5.6 **b A**
		Conventional	72 ± 15 **a C**	6.2 ± 1.7 **b A**
	2016	Organic	163 ± 50 **a B**	0.5 ± 0.2 **b A**
		Conventional	314 ± 198 **a A**	1.6 ± 0.9 **b A**
peonidin 3-*O*-glucoside (mg FW kg^−1^)	2015	Organic	325 ± 105 **a BA**	122 ± 20 **a A**
		Conventional	173 ± 50 **a B**	355 ± 98 **a B**
	2016	Organic	217 ± 29 **a B**	184 ± 24 **a A B**
		Conventional	670 ± 570 **a A**	238 ± 69 **b A B**

For each parameter assessed means labeled with the same lower case letter within the same row and same capital letters within the same column are not significantly different (General Linear Hypothesis test *p* < 0.05).

## Supplementary Materials

The following are available online at https://www.mdpi.com/2304-8158/9/12/1874/s1, Table S1: Effect of, and interaction between, production system and table grape variety on the dry matter content (DM), sugar content (SC) of pulp/juice, total phenolic content (TPC), total antioxidant activity (TAA; DPPH & TEAC assays) and total anthocyanin content (TAC) in **white** table grapes produced in South Africa from a UK supermarket survey carried out in **2015** (2-factor ANOVA, the values presented are means ± SE), Table S2: Effect of, and interaction between, production system and table grape variety on the dry matter content (DM), sugar content (SC) of pulp/juice, total phenolic content (TPC), total antioxidant activity (TAA; DPPH & TEAC assays) and total anthocyanin content (TAC) in **red** table grapes produced in South Africa from a UK supermarket survey carried out in 2015 (2-factor ANOVA, the values presented are means ± SE, Table S3: Effect of, and interaction between, production system and table grape variety on the dry matter content (DM), sugar content (SC) of pulp/juice, total phenolic content (TPC), total antioxidant activity (TAA; DPPH & TEAC assays) and total anthocyanin content (TAC) in **red** table grapes produced in Mediterranean countries from a UK supermarket survey carried out in 2015 (2-factor ANOVA, the values presented are means ± SE), Table S4: Effect of, and interaction between, production system and table grape variety on the dry matter content (DM), sugar content (SC) of pulp/juice, total phenolic content (TPC), total antioxidant activity (TAA; DPPH & TEAC assays) and total anthocyanin content (TAC) in **black** table grapes produced in Mediterranean countries from a UK supermarket survey carried out in 2015 (2-factor ANOVA, the values presented are means ± SE), Table S5: Effect of, and interaction between, production system and table grape variety on the dry matter content (DM), sugar content (SC) of pulp/juice, total phenolic content (TPC), total antioxidant activity (TAA; DPPH & TEAC assays) and total anthocyanin content (TAC) in **red** table grapes produced in Mediterranean countries from a UK supermarket survey carried out in 2016 (2-factor ANOVA, the values presented are means ± SE), Table S6: Effect of, and interaction between, production region, production system and table grape variety on the dry matter content (DM), sugar content (SC) of pulp/juice, total phenolic content (TPC), total antioxidant activity (TAA; DPPH & TEAC assays) and total anthocyanin content (TAC) in **red** table grapes from a UK supermarket survey carried out in 2015 (2-factor ANOVA, the values presented are means ± SE), Table S7: Effect of, and interaction between, year, production system and grape type on the content of individual anthocyanins in **red** table grapes produced in Mediterranean countries from a UK supermarket survey 2015 (3-factor ANOVA, the values presented are means ± SE), Table S8: Effect of, and interaction between, year, production system and grape type on the content of individual anthocyanins in **red** table grapes produced in Mediterranean countries from a UK supermarket survey 2016 (3-factor ANOVA, the values presented are means ± SE), Table S9: Effect of, and interaction between, year, production system and grape type on the content of individual anthocyanins in **black** table grapes produced in Mediterranean countries from a UK supermarket survey 2015 (3-factor ANOVA, the values presented are means ± SE). Figure S1: Principle component analyses of secondary metabolite concentration and antioxidant activity data showing the level of separation/variation between data from different (**a**) years (**b**) production regions and (**c**) production systems, Figure S2: Principle component analyses of sugar content data showing the level of separation/variation between data from different (**a**) years (**b**) production regions and (**c**) production systems, Figure S3: Principle component analyses secondary metabolite concentration and antioxidant activity data showing the separation/variation between black grape varieties and/or conventional and organic samples, Figure S4: Principle component analyses secondary metabolite concentration and antioxidant activity data showing the separation/variation between red grape varieties and/or conventional and organic samples, Figure S5: Principle component analyses secondary metabolite concentration and antioxidant activity data showing the separation/variation between black grape varieties and/or conventional and organic samples.
