# Supplementary material for: Effects of Production Region, Production Systems and Grape Type/Variety on Nutritional Quality Parameters of Table Grapes; Results from a UK Retail Survey
Source: Foods. 2020 Dec 16;9(12):1874. doi: 10.3390/foods9121874 (PMC7767105; doi:10.3390/foods9121874)
Supplement: Supplementary file 1 [file foods-09-01874-s001.pdf]

Table S1. Effect of, and interaction between, production system and table grape variety on the dry matter content (DM), sugar content (SC) of pulp/juice, total phenolic content (TPC), total antioxidant activity (TAA; DPPH & TEAC assays) and total anthocyanin content (TAC) in **white** table grapes produced in South Africa from a UK supermarket survey carried out in **2015** (2-factor ANOVA, the values presented are means ± SE)

		**Sugar content (SC)**		**Total phenolic content (TPC)**	**Antioxidant activity (TAA)**		**Total anthocyanin content (TAC)**	
**Factors**	**Dry Matter content** %	**(pulp)** Brix°	**(juice)** Brix°	mg GAE kg^{-1}	**DPPH** µmol TE g^{-1}	**TEAC** µmol TE g^{-1}	mg cyan kg^{-1}	mg mal kg^{-1}
**Production system (PS)**								
Organic (n=44)	35 ±1	16.5 ±0.4	16.3 ±0.3	1366 ±92	58 ±1	5.8 ±0.6	3.6 ±2.1	4.2 ±2.3
Conventional (n=27)	36 ±1	17.5 ±0.2	17.4 ±0.2	1173 ±60	56 ±1	5.1 ±0.4	5.4 ±2.0	6.1 ±2.2
**Variety (Vr)**								
EarlySweet (n=6)	33 ±1 **bc**	16.1 ±0.4 **bc**	16.2 ±0.4 **bc**	1279 ±95 **b**	46 ±3 **b**	6.9 ±1.2	9.1 ±3.2	11.0 ±3.3
Prime (n=15)	33 ±1 **c**	15.6 ±0.3 **c**	15.7 ±0.3 **b**	1248 ±54 **b**	55 ±2 **a**	5.3 ±0.3	4.9 ±2.1	5.9 ±2.3
Sugraone (n=22)	36 ±1 **b**	17.0 ±0.4 **b**	17.0 ±0.4 **a**	1701 ±75 **a**	60 ±1 **a**	5.0 ±0.7	3.3 ±3.5	3.9 ±3.8
Thompson (n=28)	37 ±1 **a**	18.2 ±0.2 **a**	17.8 ±0.3 **a**	882 ±46 **c**	57 ±2 **a**	5.4 ±0.6	4.8 ±2.3	5.0 ±2.4
**ANOVA (*p*-values)**								
Main effects								
PS	NS	**0.0400**	**0.0254**	**0.0108**	NS	NS	NS	NS
Vr	**0.0002**	**<0.0001**	**0.0005**	**<0.0001**	**0.0002**	NS	NS	NS
Interactions*								
PS:Vr	NS	NS	NS	**0.0110**[1]	NS	NS	NS	NS

GAE, Gallic acid equivalent; **TE**, Trolox equivalent; **cyan**, cyanidin 3-glucoside equivalent; **mal**, malvidin 3-glucoside equivalent; *p*-values in *italic* are for trends (0.1<p<0.05); * only interactions for which significant results were detected are shown; [1] see Table S1.1 for interaction means ± SE;

Table S1.1 Interactions means ± SE for the effects of grape variety and production system on the total phenolic content in table grapes.

**Parameter**	**Factor 1** **Grape Variety**	**Factor 2 Production System** Organic	Conventional
Total phenolic content (mg GAE kg^{-1})	Early Sweet	1180 ±19 **aB**	1328 ±142 **aA**
	Prime	1088 ±72 **bB**	1388 ±35 **aA**
	Sugraone	1845 ±87 **aA**	1556 ±109 **bA**
	Thompson	943 ±113 **aB**	861 ±49 **aB**

GAE, Gallic acid equivalent; For each parameter assessed means labelled with the same lower case letter within the same row and the same capital letters within the same column are not significant different (General Linear Hypothesis test *p*<0.05).

Table S2. Effect of, and interaction between, production system and table grape variety on the dry matter content (DM), sugar content (SC) of pulp/juice, total phenolic content (TPC), total antioxidant activity (TAA; DPPH & TEAC assays) and total anthocyanin content (TAC) in **red** table grapes produced in South Africa from a UK supermarket survey carried out in 2015 (2-factor ANOVA, the values presented are means ± SE

Factors	**Dry Matter content** %	**Sugar content (SC)** **(pulp)** Brix°	**(juice)** Brix°	**Total phenolic content (TPC)** mg GAE kg^{-1}	**Antioxidant activity (TAA)** **DPPH** µmol TE g^{-1}	**TEAC** µmol TE g^{-1}	**Total anthocyanin content (TAC)** mg cyan kg^{-1}	mg mal kg^{-1}
**Production System (PS)**								
Organic (n=28)	40 ±1	18.8 ±0.3	19.1 ±0.3	1777 ±75	96 ±5	8.5 ±1.1	99 ±10	104 ±11
Conventional (n=29)	39 ±1	18.4 ±0.3	18.3 ±0.3	1471 ±73	88 ±4	5.3 ±0.7	120 ±12	127 ±13
**Variety (Vr)**								
Allison (n=8)	40 ±1	18.5 ±0.4	18.9 ±0.4 **ab**	1845 ±63 **a**	100 ±3 **ab**	4.6 ±0.3 **b**	126 ±28	133 ±30
Crimson (n=25)	39 ±1	18.7 ±0.3	18.6 ±0.3 **ab**	1325 ±44 **b**	96 ±1 **b**	6.2 ±0.7 **b**	117 ±14	124 ±14
Flame (n=10)	37 ±1	17.6 ±0.5	17.7 ±0.4 **b**	1903 ±184 **a**	52 ±1 **c**	2.7 ±0.3 **b**	96 ±16	101 ±17
Sweet Celebration (n=14)	41 ±1	19.3 ±0.5	19.4 ±0.4 **a**	1821 ±92 **a**	109 ±5 **a**	12.0 ±1.7 **a**	96 ±11	101 ±12
**ANOVA (*p*-values)**								
Main effects								
PS	NS	NS	**0.0407**	**0.0038**	**0.0166**	**0.0113**	NS	NS
Vr	NS	NS	NS	**0.0008**	**<.0001**	**0.0001**	NS	NS
Interactions*								
PS:Vr	NS	NS	NS	NS	NS	NS	NS	NS

GAE, Gallic acid equivalent; **TE**, Trolox equivalent; **cyan**, cyanidin 3-glucoside equivalent; **mal**, malvidin 3-glucoside equivalent; *p*-values in *italic* are for trends (0.1<p<0.05); only interactions for which significant results were detected are shown;

Table S3. Effect of, and interaction between, production system and table grape variety on the dry matter content (DM), sugar content (SC) of pulp/juice, total phenolic content (TPC), total antioxidant activity (TAA; DPPH & TEAC assays) and total anthocyanin content (TAC) in **red** table grapes produced in Mediterranean countries from a UK supermarket survey carried out in 2015 (2-factor ANOVA, the values presented are means ± SE)

**Factors**	**Dry Matter content** %	**Sugar content (SC)**		**Total phenolic content (TPC)**	**Antioxidant activity (TAA)**		**Total anthocyanin content (TAC)**	
		**(pulp)** Brix°	**(juice)** Brix°	mg GAE kg-	**DPPH** μmol TE g-1	**TEAC** μmol TE g-1	mg cyan kg-1	mg mal kg-1
**Production system (PS)**								
ORG (n=30)	37 ±1	17.7 ±0.3	17.9 ±0.3	2170 ±141	110 ±6	9.3 ±0.9	62 ±7	66 ±8
CON (n=30)	37 ±1	17.5 ±0.2	17.9 ±0.3	1955 ±109	111 ±7	9.5 ±1.1	101 ±17	106 ±18
**Variety (Vr)**								
Allison (n=4)	37 ±2	17.7 ±0.8 **ab**	18.5 ±0.7 **ab**	1913 ±288 **ab**	99 ±5 **b**	9.0 ±1.7 **ab**	83 ±31	88 ±33
Crimson (n=33)	37 ±1	17.1 ±0.2 **b**	17.5 ±0.2 **b**	1930 ±80 **b**	105 ±4 **b**	9.1 ±0.7 **b**	81 ±11	86 ±12
Flame (n=10)	33 ±4	17.8 ±0.4 **ab**	18.0 ±0.5 **ab**	2640 ±276 **a**	151 ±16 **a**	15.0 ±2.8 **a**	85 ±9	90 ±9
Scarlotta (n=13)	38 ±1	18.5 ±0.5 **a**	18.7 ±0.4 **a**	2005 ±250 **b**	96 ±4 **b**	6.1 ±0.6 **b**	79 ±34	83 ±36
**ANOVA (*p*-values)**								
Main effects								
PS	NS	NS	NS	NS	NS	NS	NS	NS
Vr	NS	**0.0284**	**0.0448**	**0.0338**	**0.0008**	**0.0028**	NS	NS
Interactions *								
PS:Vr	NS	**0.0354**[1]	NS	NS	NS	NS	NS	NS

GAE, Gallic acid equivalent; **TE**, Trolox equivalent; **cyan**, cyanidin 3-glucoside equivalent; **mal**, malvidin 3-glucoside equivalent; *p*-values in *italic* are for trends (0.1<p<0.05); * only interactions for which significant results were detected are shown; [1] see Table S3.1 for interaction means ± SE;

Table S3.1 Interactions means ± SE for the effects of grape type and production system on the sugar content in table grapes.

**Parameter**	**Factor 1** **Grape Variety**	**Factor 2 Production System** Organic	Conventional
Sugar content (**pulp** Brix°)	Allison	18.7 ±0.9 **aA**	16.7 ±1.2 **aA**
	Crimson	16.7 ±0.3 **bB**	17.4 ±0.3 **aA**
	Flame	18.4 ±0.3 **aA**	17.3 ±0.6 **aA**
	Scarlotta	18.4 ±0.6 **aA**	18.8 ±0.5 **aB**

For each parameter assessed means labelled with the same lower case letter within the same row and the same capital letters within the same column are not significant different (General Linear Hypothesis test *p*<0.05).

Table S4. Effect of, and interaction between, production system and table grape variety on the dry matter content (DM), sugar content (SC) of pulp/juice, total phenolic content (TPC), total antioxidant activity (TAA; DPPH & TEAC assays) and total anthocyanin content (TAC) in **black** table grapes produced in Mediterranean countries from a UK supermarket survey carried out in 2015 (2-factor ANOVA, the values presented are means ± SE)

Factors	Dry Matter content %	Sugar content (SC) (pulp) Brix°	(juice) Brix°	Total phenolic content (TPC) mg GAE kg-	Antioxidant activity (TAA) DPPH µmol TE g-1	TEAC µmol TE g-1	Total anthocyanin content (TAC) mg cyan kg-1	mg mal kg-1
**Production system (PS)**								
Organic (n=9)	34 ±2	16.5 ±0.8	17.3 ±0.9	2746 ±316	139 ±11	24 ±3	552 ±133	582 ±140
Conventional (n=11)	34 ±1	16.4 ±0.7	16.5 ±0.6	2250 ±232	117 ±10	16 ±2	426 ±60	450 ±63
**Variety (Vr)**								
Autumn Royal (n=8)	33 ±1	15.0 ±0.5b	15.5 ±0.6 **b**	2069 ±362	109 ±5 **b**	15 ±2 **b**	237 ±54 **b**	250 ±57 **b**
Midnight Beauty (n=12)	36 ±2	17.5 ±0.6a	17.8 ±0.7 **a**	2742 ±190	139 ±11 **a**	23 ±3 **a**	646 ±77 **a**	682 ±81 **a**
**ANOVA (*p*-values)**								
Main effects								
PS	NS	NS	NS	NS	NS	**0.0498**	NS	NS
Vr	NS	**0.0027**	**0.0044**	NS	**0.0321**	**0.0291**	**0.0028**	**0.0028**
Interactions*								
PS:Vr	NS	NS	**0.0026**[1]	NS	NS	NS	**0.0307**[1]	**0.0307**[1]

GAE, Gallic acid equivalent; **TE**, Trolox equivalent; **cyan**, cyanidin 3-glucoside equivalent; **mal**, malvidin 3-glucoside equivalent; *p*-values in *italic* are for trends (0.1<p<0.05); * only interactions for which significant results were detected are shown; [1] see Table S5.1 for interaction means ± SE;

Table S4.1 Interactions means ± SE for the effects of grape variety and production systems on the total anthocyanin content and sugar content in table grapes.

Parameter	Factor 1 Grape Variety	Factor 2 Production System Organic	Conventional
Sugar content (**juice** Brix°)	Autumn Royal	14.6 ±0.7 **aA**	16.4 ±0.8 **aA**
	Midnight Beauty	19.4 ±0.4 **bB**	16.6 ±0.9 **aA**
Total anthocyanin content (mg cyan kg-1)	Autumn Royal	177 ±36 **aA**	297 ±99 **aA**
	Midnight Beauty	851 ±110 **aB**	499 ±64 **bA**
Total anthocyanin content (mg mal kg-1)	Autumn Royal	187 ±38 **aB**	313 ±104 **aA**
	Midnight Beauty	898 ±116 **aA**	528 ±67 **bA**

cyan, cyanidin 3-glucoside equivalent; mal, malvidin 3-glucoside equivalent; For each parameter assessed means labelled with the same lower case letter within the same row and the same capital letters within the same column are not significant different (General Linear Hypothesis test *p*<0.05).

Table S5. Effect of, and interaction between, production system and table grape variety on the dry matter content (DM), sugar content (SC) of pulp/juice, total phenolic content (TPC), total antioxidant activity (TAA; DPPH & TEAC assays) and total anthocyanin content (TAC) in **red** table grapes produced in Mediterranean countries from a UK supermarket survey carried out in 2016 (2-factor ANOVA, the values presented are means ± SE)

**Factors**	**Dry Matter content** %	**Sugar content (SC)** **(pulp)** Brix°	**(juice)** Brix°	**Total phenolic content (TPC)** mg GAE kg-	**Antioxidant activity (TAA)** **DPPH** μmol TE g^{-1}	**TEAC** μmol TE g^{-1}	**Total anthocyanin content (TAC)** mg cyan kg^{-1}	mg mal kg^{-1}
**Production system (PS)**								
Organic (n=7)	40 ±2	18.8 ±0.6	19.1±0.5	2033 ±139	142 ±3	5.4±0.6	78 ±8	82 ±9
Conventional (n=7)	40 ±2	18.5 ±1.0	19.2±0.7	2230 ±148	140 ±1	5.8±0.4	175 ±43	184 ±46
**Variety (Vr)**								
Allison (n=5)	40 ±2	18.3 ±0.5	18.8±0.5	2058 ±140	143 ±2	6.4±0.4	127 ±41	134 ±43
Crimson (n=9)	40 ±2	18.8 ±0.8	19.3±0.6	2172 ±141	140 ±2	5.2±0.5	126 ±34	133 ±35
**ANOVA (p-values)**								
Main effects								
PS	NS	NS	NS	NS	NS	NS	NS	NS
Vr	NS	NS	NS	NS	NS	NS	NS	NS
Interactions*								
PS:Vr	NS	NS	NS	NS	NS	NS	NS	NS

GAE, Gallic acid equivalent; **TE**, Trolox equivalent; **cyan**, cyanidin 3-glucoside equivalent; **mal**, malvidin 3-glucoside equivalent; *p*-values in *italic* are for trends (0.1<p<0.05); * only interactions for which significant results were detected are shown;

Table S6. Effect of, and interaction between, production region, production system and table grape variety on the dry matter content (DM), sugar content (SC) of pulp/juice, total phenolic content (TPC), total antioxidant activity (TAA; DPPH & TEAC assays) and total anthocyanin content (TAC) in **red** table grapes from a UK supermarket survey carried out in 2015 (2-factor ANOVA, the values presented are means ± SE)

Factors	Dry Matter content %	Sugar content (SC) (pulp) Brix°	(juice) Brix°	Total phenolic content (TPC) mg GAE kg^{-1}	Antioxidant activity (TAA) DPPH µmol TE g^{-1}	TEAC µmol TE g^{-1}	Total anthocyanin content (TAC) mg cyan kg^{-1}	mg mal kg^{-1}
**Production region (PR)**								
South Africa (n=35)	39 ±1	18.4 ±0.2	18.4 ±0.2	1490 ±74	83 ±4	5.2 ±0.5	111 ±11	117 ±11
Mediterranean (n=43)	36 ±1	17.3 ±0.2	17.6 ±0.2	2095 ±98	115 ±6	10.5 ±0.9	82 ±9	87 ±9
**Production system (PS)**								
Organic (n=28)	37 ±1	17.7 ±0.3	18.0 ±0.3	2030 ±129	104 ±7	9.3 ±1.1	81 ±8	86 ±8
Conventional (n=50)	38 ±1	17.8 ±0.2	17.9 ±0.2	1708 ±82	100 ±5	7.4 ±0.8	103 ±10	109 ±10
**Variety (Vr)**								
Crimson (n=58)	38 ±1	17.8 ±0.2	18.0 ±0.2	1669 ±63	101 ±3	7.8 ±0.5	97 ±9	102 ±9
Flame **(n=20)**	35 ±2	17.7 ±0.3	17.8 ±0.3	2272 ±182	101 ±14	8.9 ±2.0	91 ±9	96 ±10
**ANOVA (*p*-values)**								
Main effects								
PR	**0.0319**	**0.0017**	**0.0157**	**<0.0001**	**0.0001**	**0.0003**	**0.0425**	**0.0425**
PS	NS	NS	NS	NS	NS	NS	NS	NS
Vr	**0.0312**	NS	NS	**<0.0001**	NS	NS	NS	NS
Interactions*								
PR : PS	NS	NS	**0.0219**[1]	NS	NS	NS	NS	NS
PR : Vr	NS	**0.0127**[2]	**0.0228**[2]	NS	**<0.0001**[2]	**0.0004**[2]	NS	NS
PS : Vr	NS	NS	NS	NS	NS	NS	**0.0182**[3]	**0.0182**[3]
PR : PS : Vr	NS	NS	**0.0442**[4]	NS	NS	NS	NS	NS

GAE, Gallic acid equivalent; **TE**, Trolox equivalent; **cyan**, cyanidin 3-glucoside equivalent; **mal**, malvidin 3-glucoside equivalent; *p*-values in *italic* are for trends (0.1<p<0.05); * only interactions for which significant results were detected are shown; [1] see Table S6.1 for interaction means ± SE; [2] see Table S6.2 for interaction means ± SE; [3] see Table S6.3 for interaction means ± SE; [4] see Table S6.4 for interaction means ± SE;

Table S6.1 Interactions means ± SE for the effects of production system and season on the sugar content in table grapes.

Parameter	Factor 1	Factor 2 Production System	
	**Production region**	Organic	Conventional
Sugar content (**juice** Brix°)	South Africa	19.2 ±0.4 **aA**	18.0 ±0.3 **bA**
	Mediterranean	17.4 ±0.3 **aB**	17.7 ±0.3 **aA**

For each parameter assessed means labelled with the same lower case letter within the same row and the same capital letters within the same column are not significant different (General Linear Hypothesis test $p<0.05$).

Table S6.2 Interactions means ± SE for the effects of grape variety and season on the sugar content and antioxidant activity (TEAC, DPPH) in table grapes.

	Factor 1	Factor 2 Production region	
**Parameter**	**Grape Variety**	Mediterranean	South Africa
Sugar content (**pulp** Brix°)	Crimson	17.1 ±0.2 **bA**	18.6 ±0.3 **aA**
	Flame	17.8 ±0.4 **aA**	17.6 ±0.5 **aA**
Sugar content (**juice** Brix°)	Crimson	17.5 ±0.2 **aA**	18.6 ±0.3 **bA**
	Flame	17.9 ±0.5 **aA**	17.7±0.4 **aA**
Antioxidant activity (**TEAC,** μmol TE g^{-1})	Crimson	9.1 ±0.7 **aB**	6.7 ±0.7 **bA**
	Flame	15.0 ±2.8 **aA**	2.7 ±0.3 **bA**
Antioxidant activity (**DPPH,** μmol TE g^{-1})	Crimson	104 ±4 **aB**	96 ±1 **bA**
	Flame	151 ±16 **aA**	52 ±1 **bB**

For each parameter assessed means labelled with the same lower case letter within the same row and the same capital letters within the same column are not significant different (General Linear Hypothesis test $p<0.05$).

Table S6.3 Interactions means ± SE for the effects of grape variety and production system on the total anthocyanin content in table grapes.

Parameter	Factor 1	Factor 2 Production System	
	**Grape Variety**	Organic	Conventional
Total anthocyanin content (mg cyan kg^{-1})	Crimson	68 ±9 **bA**	110 ±12 **aA**
	Flame	108 ±15 **aA**	76 ±10 **aB**
Total anthocyanin content (mg mal kg^{-1})	Crimson	72 ±9 **bA**	116 ±12 **aA**
	Flame	115 ±16 **aA**	80 ±10 **aB**

cyan, cyanidin 3-glucoside equivalent; **mal**, malvidin 3-glucoside equivalent; For each parameter assessed means labelled with the same lower case letter within the same row and the same capital letters within the same column are not significant different (General Linear Hypothesis test $p<0.05$).

Table S6.4 Interactions means ± SE for the effects of production system, season and grape variety on the sugar content in table grapes.

Parameter	Factor 1	Factor 2	Factor 3 Production System	
	**Production region**	**Grape Variety**	Organic	Conventional
Sugar content (**juice** Brix°)	Mediterranean	Crimson	16.9 ±0.3 **aB**	17.8 ±0.3 **aA**
		Flame	18.5 ±0.5 **aA**	17.5 ±0.8 **aA**
	South Africa	Crimson	19.9 ±0.3 **aA**	18.2 ±0.3 **bA**
		Flame	18.1 ±0.3 **aA**	17.4 ±0.7 **aA**

For each parameter assessed means labelled with the same lower case letter within the same row and the same capital letters within the same column are not significant different (General Linear Hypothesis test $p<0.05$).

Table S7. Effect of, and interaction between, year, production system and grape type on the content of individual anthocyanins in **red** table grapes produced in Mediterranean countries from a UK supermarket survey 2015 (3-factor ANOVA, the values presented are means ± SE).

Factor	Concentrations of individual anthocyanins (mg FW kg^{-1})						
	delphinidin 3-O-glucoside	cyanindin 3-O-glucoside	petunidin 3-O-glucoside	peonidin 3-O-glucoside	malvidin 3-O-glucoside	peonidin 3-O-p-coumaroyl glucoside	malvidin 3-O-p-coumaroyl glucoside
**Production system (PS)**							
Organic (n=10)	14.7 ±6.9	26 ±11	12.5 ±5.6	122 ±20	47 ±15	5 ±2	1.4 ±0.4
Conventional (n=9)	5.9 ±2.1	32 ±10	6.8 ±1.8	378 ±106	60 ±13	8 ±3	2.3 ±0.8
**Variety (Vr)**							
Crimson (n=11)	0.6 ±0.2	9 ±2	1.6 ±0.5	318 ±95	40 ±14	8 ±2	2.0 ±0.7
Flame (n=8)	24.2 ±6.7	57 ±11	21.1 ±4.9	141 ±24	70 ±13	6 ±2	1.5 ±0.4
**ANOVA *p*-values**							
Main effects							
PS	NS	NS	NS	**0.0092**	NS	NS	NS
Vr	**0.0001**	**0.0001**	**<0.0001**	NS	NS	NS	NS
Interactions*							
PS : Vr	**0.0156**[1]	NS	**0.0104**[1]	**0.0335**[1]	**0.0044**[1]	**0.0216**[1]	**0.0078**[1]

p-values in *italic* are for trends (0.1<p<0.05); * only interactions for which significant results were detected are shown; [1] see Table S7.1 for interaction means ± SE.

Table S7.1 Interactions means ± SE for the effects of grape variety and production system on the concentrations of individual anthocyanin compounds in red grapes produced in the Mediterranean (2015).

Parameter	Factor 1 Variety	Factor 2 Production system	
		Organic	Conventional
delphinidin 3-O-glucoside (mg FW kg^{-1})	Crimson	0.4 ±0.2 **a B**	0.9 ±0.4 **a A**
	Flame	36.2 ±10.5 **a A**	12.2 ±1.7 **b A**
petunidin 3-O-glucoside (mg FW kg^{-1})	Crimson	0.8 ±0.4 **a B**	2.6 ±0.9 **a B**
	Flame	30.2 ±7.8 **a A**	12.1 ±1.2 **b A**
peonidin 3-O-glucoside (mg FW kg^{-1})	Crimson	120 ±20 **b A**	555 ±151 **a A**
	Flame	126 ±45 b **A**	157 ±24 a **B**
malvidin 3-O-glucoside (mg FW kg^{-1})	Crimson	15 ±5 **b A**	71 ±23 **a A**
	Flame	96 ±19 **a A**	46 ±5 **b A**
peonidin 3-O-p-coumaroyl glucoside (mg FW kg^{-1})	Crimson	3.5 ±1.1 **b A**	12.7 ±3.9 **a A**
	Flame	8.3 ±4.5 **a A**	3.1 ±0.7 **a B**
malvidin 3-O-p-coumaroyl glucoside (mg FW kg^{-1})	Crimson	0.7 ±0.3 **b A**	3.6 ±1.2 **a A**
	Flame	2.3 ±0.7 **a A**	0.6 ±0.1 **a B**

For each parameter assessed means labeled with the same lower case letter within the same row and same capital letters within the same column are not significant different (General Linear Hypothesis test *p*<0.05).

Table S8. Effect of, and interaction between, year, production system and grape type on the content of individual anthocyanins in **red** table grapes produced in Mediterranean countries from a UK supermarket survey 2016 (3-factor ANOVA, the values presented are means ± SE).

Factor	Concentrations of individual anthocyanins (mg FW kg^{-1})						
	delphini-din 3-O- glucoside	cyanin-din 3-O-glucoside	petuni-din 3-O-glucoside	peoni -din 3-O-glucoside	malvi-din 3-O-glucoside	peonidin 3-O-p-coumaroyl glucoside	malvidin 3-O-p-coumaroyl glucoside
**Production system (PS)**							
Organic (n=5)	0.2 ±0.1	8 ±3	0.5 ±0.2	184 ±24	20 ±8	13 ±7	2.4 ±1.4
Conventional (n=5)	0.8 ±0.5	28 ±13	1.6 ±0.9	238 ±69	24 ±8	9 ±4	0.9 ±0.3
**Variety (Vr)**							
Crimson (n=6)	0.2 ±0.1	36 ±14	1.9 ±1.1	300 ±52	29 ±10	25 ±5	3.5 ±1.5
Allison (n=4)	1.1 ± 0.7	6 ±1	0.5 ±0.2	152 ±30	17 ±5	2 ±1	0.4 ±0.2
**ANOVA *p*-values**							
Main effects							
PS	NS	**0.0033**	NS	NS	NS	NS	NS
Vr	NS	NS	NS	NS	NS	NS	NS
Interactions*							
PS : Vr	NS	**0.0033**[1]	NS	**0.0106**[1]	NS	**0.0442**[1]	**0.0144**[1]

p-values in *italic* are for trends (0.1<p<0.05); * only interactions for which significant results were detected are shown; [1] see Table S8.1 for interaction means ± SE.

Table S8.1 Interactions means ± SE for the effects of grape variety and production system on the concentrations of individual anthocyanin compounds in red grapes produced in the Mediterranean (2016).

Parameter	Factor 1	Factor 2 Production system	
	Variety	Organic	Conventional
cyanindin 3-O-glucoside (mg FW kg^{-1})	Crimson	4 ±1 **a A**	7 ±2 **a B**
	Allison	13 ±5 **b A**	58 ±8 **a A**
peonidin 3-O-glucoside (mg FW kg^{-1})	Crimson	163 ±30 **a A**	140 ±59 **a B**
	Allison	214 ±37 **b A**	386 ±6 **a A**
peonidin 3-O-p-coumaroyl glucoside (mg FW kg^{-1})	Crimson	1.4 ±0.2 **a B**	2.8 ±1.4 **a B**
	Allison	31.0 ±4.4 **a A**	18.5 ±4.8 **b A**
malvidin 3-O-p-coumaroyl glucoside (mg FW kg^{-1})	Crimson	0.2 ±0.0 **a B**	0.6 ±0.3 **a A**
	Allison	5.7 ±1.7 **a A**	1.3 ±0.4 **b A**

For each parameter assessed means labeled with the same lower case letter within the same row and same capital letters within the same column are not significant different (General Linear Hypothesis test *p*<0.05).

Table S9. Effect of, and interaction between, year, production system and grape type on the content of individual anthocyanins in **black** table grapes produced in Mediterranean countries from a UK supermarket survey 2015 (3-factor ANOVA, the values presented are means ± SE).

Factor	Concentrations of individual anthocyanins (mg FW kg^{-1})						
	delphinidin 3-O-glucoside	cyanindin 3-O-glucoside	petunidin 3-O-glucoside	peonidin 3-O-glucoside	malvidin 3-O-glucoside	peonidin 3-O-p-coumaroyl glucoside	malvidin 3-O-p-coumaroyl glucoside
**Production system (PS)**							
Organic (n=8)	74 ±27	19 ±11	132 ±39	324 ±105	1334 ±241	70 ±14	534 ±90
Conventional (n=9)	32 ±8	14 ±10	72 ±15	172 ±50	1033 ±156	51 ±12	605 ±172
**Variety (Vr)**							
Autumn royal (n=8)	34 ±7	19 ±11	68 ±9	258 ±37	935 ±94	68 ±11	465 ±90
Midnight beauty (n=9)	68 ±25	14 ±10	130 ±37	233 ±107	1387 ±236	53 ±14	666 ±166
**ANOVA *p*-values**							
Main effects							
PS	NS	NS	NS	NS	NS	NS	NS
Vr	NS	NS	NS	NS	NS	NS	NS
Interactions*							
PS : Vr	NS	NS	NS	**0.0278**[1]	NS	NS	NS

p-values in *italic* are for trends (0.1<p<0.05); * only interactions for which significant results were detected are shown; [1] see Table S9.1 for interaction means ± SE.

Table S9.1 Interactions means ± SE for the effects of grape variety and production system on the concentrations of individual anthocyanin compounds in red grapes produced in the Mediterranean (2016).

Parameter	Factor 1	Factor 2 Production system	
	**Variety**	**Organic**	**Conventional**
Peonidin 3-O- glucoside (mg FW kg^{-1})	Autumn royal	201 ±35 **aB**	314 ±55 **aA**
	Midnight beauty	449 ±201 **aA**	60 ±9 **bA**

For each parameter assessed means labeled with the same lower case letter within the same row and same capital letters within the same column are not significant different (General Linear Hypothesis test *p*<0.05).

a.

b.

c.

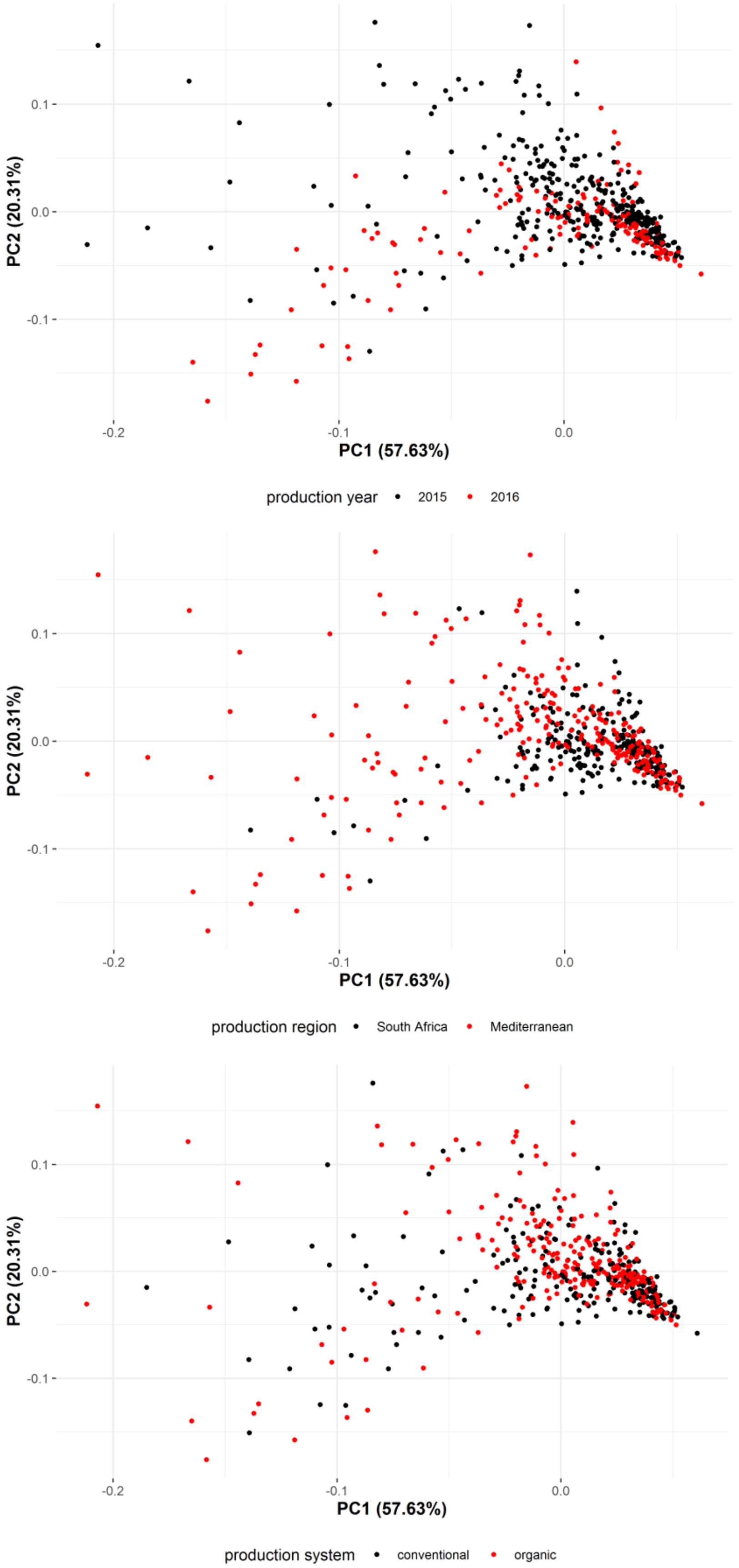

Figure S1. Principle component analyses of secondary metabolite concentration and antioxidant activity data showing the level of separation/variation between data from different **(a)** years **(b)** production regions and **(c)** production systems

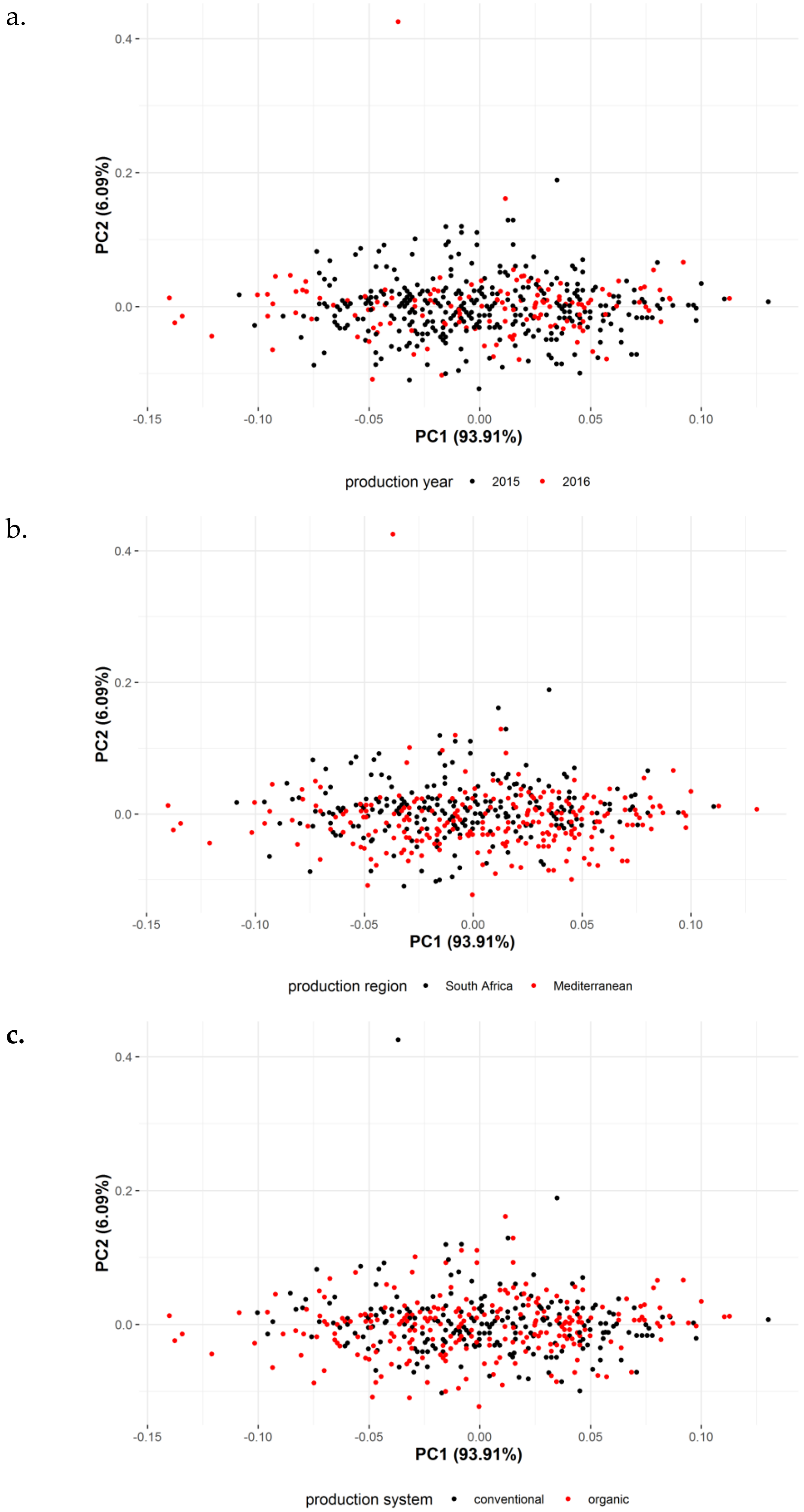

Figure S2. Principle component analyses of sugar content data showing the level of separation/variation between data from different **(a)** years **(b)** production regions and **(c)** production systems

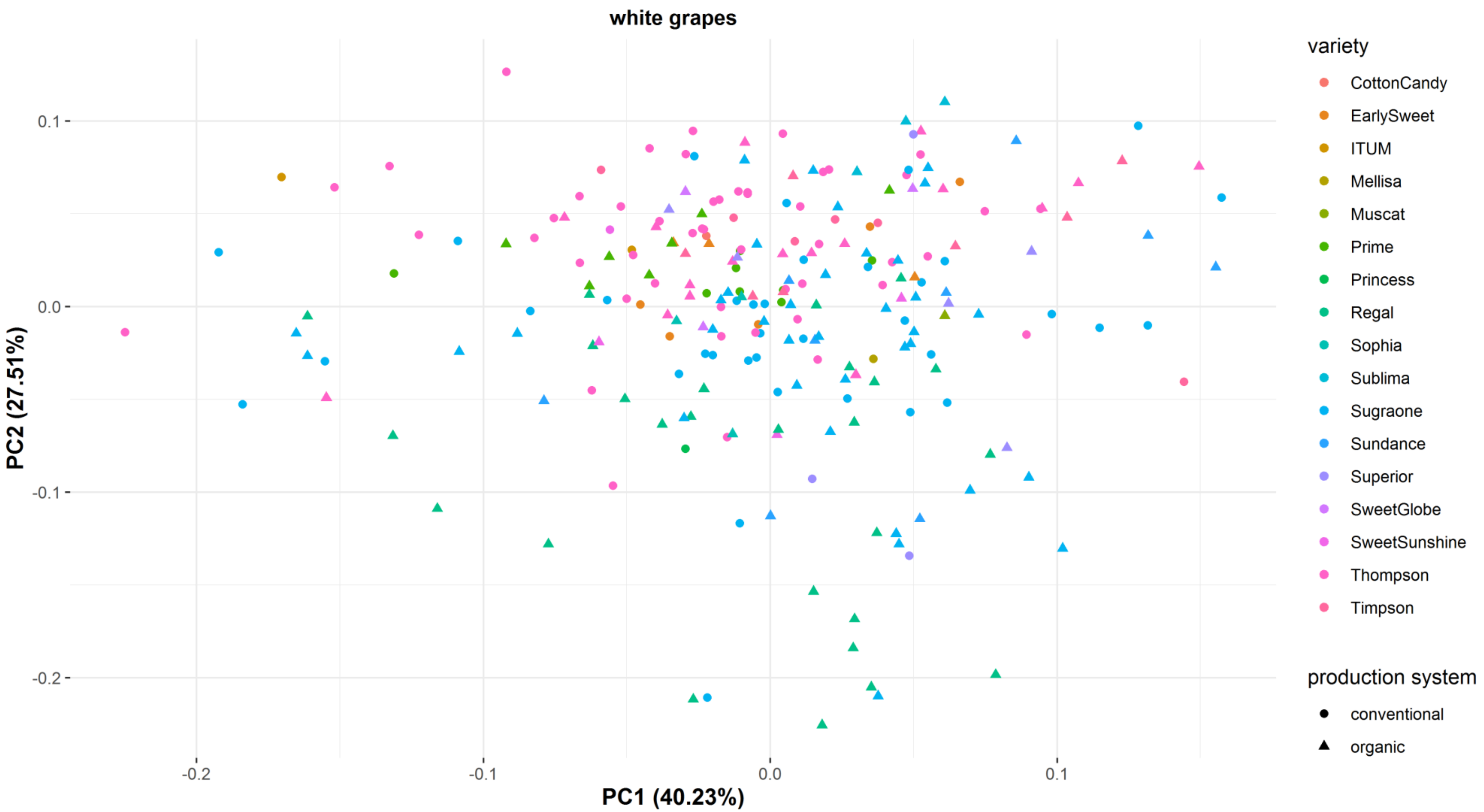

Figure S3. Principle component analyses secondary metabolite concentration and antioxidant activity data showing the separation/variation between black grape varieties and/or conventional and organic samples.

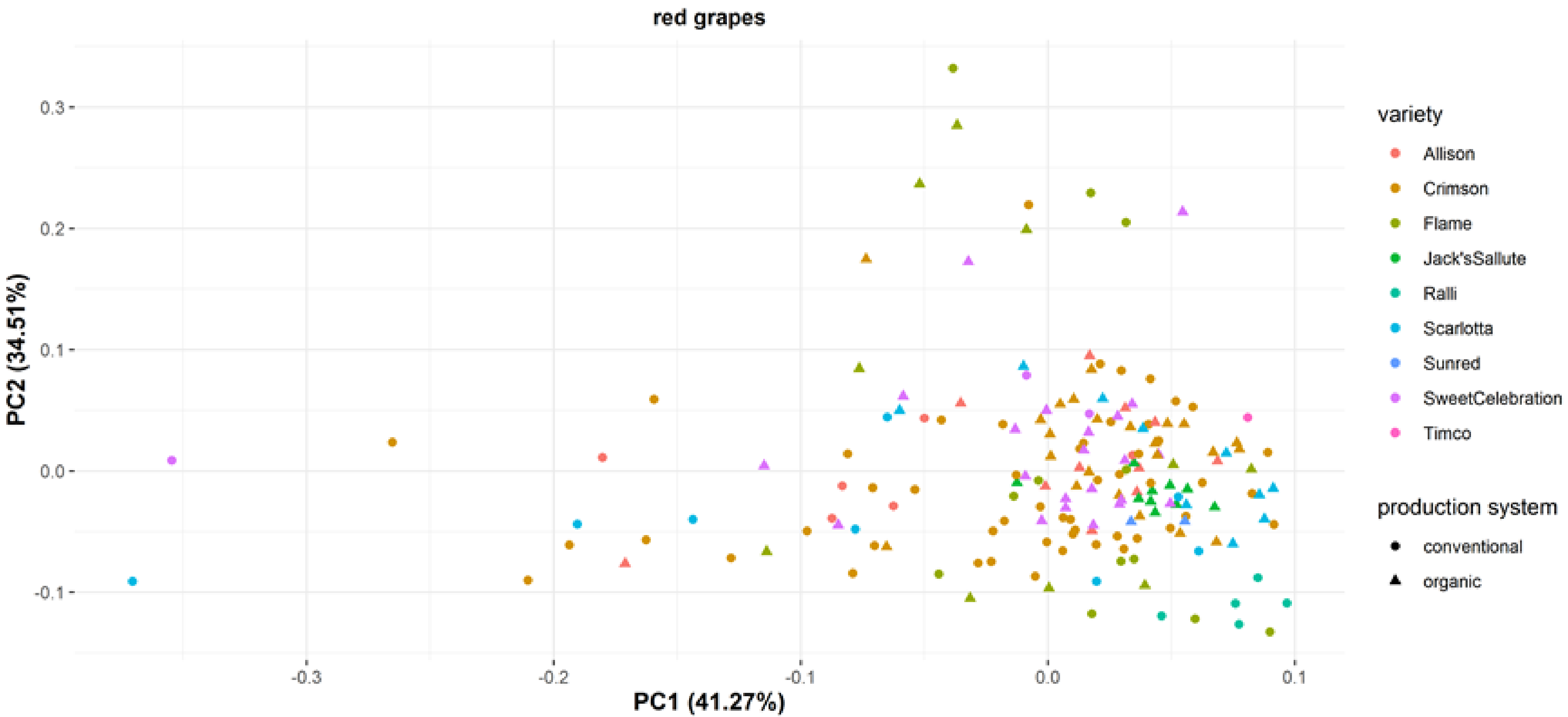

Figure S4. Principle component analyses secondary metabolite concentration and antioxidant activity data showing the separation/variation between red grape varieties and/or conventional and organic samples.

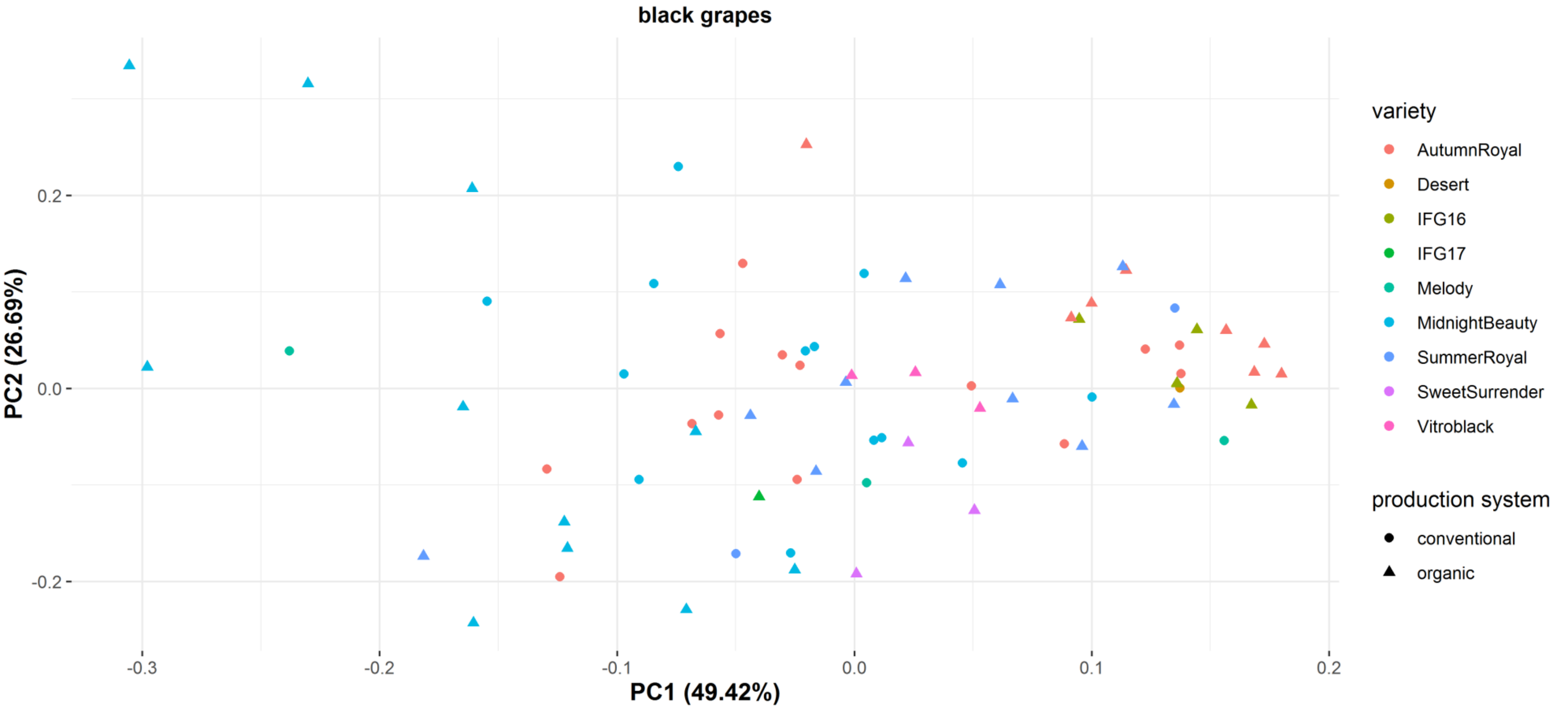

Figure S5. Principle component analyses secondary metabolite concentration and antioxidant activity data showing the separation/variation between black grape varieties and/or conventional and organic samples.
